# Relationship between Macroeconomic Indicators and Economic Cycles in U.S

**DOI:** 10.1038/s41598-020-65002-3

**Published:** 2020-05-21

**Authors:** Hiroshi Iyetomi, Hideaki Aoyama, Yoshi Fujiwara, Wataru Souma, Irena Vodenska, Hiroshi Yoshikawa

**Affiliations:** 1 Niigata University, Department of Mathematics, Niigata, 950-2181 Japan; 2 Kyoto University, GSAIS, Kyoto, 606-8502 Japan; 3 Research Institute of Economy, Trade and Industry (RIETI), Tokyo, 100-8901 Japan; 4 University of Hyogo, Graduate School of Simulation Studies, Kobe, 650-0047 Japan; 5 Nihon University, College of Science and Technology, Funabashi, 274-8501 Japan; 6 Boston University, Metropolitan College, Boston, MA 02215 USA; 7 Rissho University, Faculty of Economics, Tokyo, 141-8602 Japan

**Keywords:** Complex networks, Scientific data, Statistics

## Abstract

We analyze monthly time series of 57 US macroeconomic indicators (18 leading, 30 coincidental, and 9 lagging) and 5 other trade/money indexes. Using novel methods, we confirm statistically significant co-movements among these time series and identify noteworthy economic events. The methods we use are Complex Hilbert Principal Component Analysis (CHPCA) and Rotational Random Shuffling (RRS). We obtain significant complex correlations among the US economic indicators with leads/lags. We then use the Hodge decomposition to obtain the hierarchical order of each time series. The Hodge potential allows us to better understand the lead/lag relationships. Using both CHPCA and Hodge decomposition approaches, we obtain a new lead/lag order of the macroeconomic indicators and perform clustering analysis for positively serially correlated positive and negative changes of the analyzed indicators. We identify collective negative co-movements around the Dot.com bubble in 2001 as well as the Global Financial Crisis (GFC) in October 2008. We also identify important events such as the Hurricane Katrina in August 2005 and the Oil Price Crisis in July 2008. Additionally, we demonstrate that some coincidental and lagging indicators actually show leading indicator characteristics. This suggests that there is a room for existing indicators to be improved.

## Introduction

“During six weeks in late 1937, Wesley Mitchell, Arthur Burns, and their colleagues at the National Bureau of Economic Research developed a list of leading, coincident, and lagging indicators of economic activity in the United States as part of the NBER research program on business cycles. Since their development, these indicators, in particular the leading and coincident indexes constructed from these indicators, have played an important role in summarizing and forecasting the state of macro-economic activity”^1^.

Business cycles are important. Economists and policymakers closely follow macroeconomic indicators, especially leading ones meant to precede business cycles, to discern whether we could expect expansions or contractions in the near future.

Given that there are many different causes of cyclical business expansions and contractions, there are also different symptoms or early warning indicators for economic upturns or downturns. Some macroeconomic indicators may perform better in specific periods, while others may be more suitable for forecasting business cycles in other sets of conditions. Some of the criteria for indicators performance include: (1) economic significance; (2) statistical adequacy; and (3) consistency^2^. There have been numerous revisions of historical lists of leading economic indicators since the 1930s, when the first list was created by Mitchell and Burns^3^ at the National Bureau of Economic Research (NBER). Based on a study of approximately 500 macroeconomic indicators, they identified 21 indicators as most trustworthy. A follow-up study conducted by Moore^4^ investigated 800 indicators, and in 1961, the first composite indicator of leading indicators was created^5^. In 1967, Moore and Shiskin introduced a specific scoring system for an evaluation of one hundred time series^6^. In the early 1970s, in the midst of two recessions (1970 and 1974), NBER and the Bureau of Economic Analysis (BEA) jointly worked on revisions of the nominal indicators to account for high inflation^7^.

In 1989, an interesting approach to construct macroeconomic indicators was built by Stock and Watson^1^. Their assumption was that the co-movements of the economic time series are related to an unobserved variable called “state of the economy.” The Stock and Watson leading indicator is very different from the older NBER and BEA indexes. It is based on a VAR model with seven selected leading variables, mostly focusing on interest rates and interest rate spreads, building permits, durable goods orders, and part-time work in non-agricultural industries^1^.

The Stock and Watson indicator was retired after 14 years of existence (1989–2003), monthly measurements, and reports. Based on newer methods for assessing the current state of the economy and considerable advancements in measurements of business cycle trends, research has produced the Chicago Fed National Activity Index (CFNAI), which is also the most direct replacement of the Stock-Watson indexes. CFNAI is a monthly index, constructed from 85 monthly indicators based on an extension of the methodology used to construct the original Stock-Watson indexes. Economic activity usually has a trend tendency in growth/decline over time, and a positive value in the CFNAI signifies growth above the trend, while a negative value of the index corresponds to a growth below the trend.

In 2011, researchers at the Conference Board proposed a structural change in the Leading Economic Index (LEI), replacing three components: (1) incorporating a Leading Credit Index (LEI) instead of real money supply M2; (2) replacing ISM Supplier Delivery index with ISM New Order Index; and (3) changing the Reuters/University of Michigan Consumer Expectation Index by a weighted average of consumer expectations based on surveys administered by the Conference Board and Reuters/University of Michigan^8^. These Conference Board changes, along with research reports indicating varying roles of macroeconomic indicators in their relationships with the business cycle, opened many interesting questions and motivated analysis of the current leading, coincident, and lagging indexes to improve forecasting of economic activity.

Sophisticated forecasting techniques have been used to infer the direction of the economy based on analysis of diffusion indexes^9^. Though sophisticated, they were based largely on trial and error. The principal component analysis (PCA)^10^ provides a systematic method for defining indicators, but it suffers from two shortcomings: (1) Correlation with time leads and lags. In most cases, change in one variable may affect other variables with time delay, which results in correlation with time lead/lag. PCA, in its simplest form, studies equal-time correlation. Therefore, one needs to time-shift variables to each other and seek to maximize the correlation coefficients as functions of the amount of the time-shifts. This is complicated and requires a great deal of computing resources when one has a large number of time series. (2) How to identify statistically meaningful ones out of all the eigenmodes of the correlation matrix. There are few established significance tests for PCA. Random Matrix Theory (RMT) provides a systematic method for testing significance of eigenmodes, but it critically depends on the requirement that the number of times series is “sufficiently” large and all the autocorrelations are trivial. This paper presents a novel method for overcoming the shortcomings of standard PCA and introduces a novel analytical framework for studying macroeconomic indicators and assessing their leading role in forecasting business cycle turning points.

The rest of this paper is organized as follows. First, in the data section, we describe the data used in our empirical analysis. Then, in the methods section, we offer a detailed explanation of our methodologies. In the results section, we describe our results. The final section offers concluding remarks.

## Data

We analyze 57 macroeconomic indicators (18 Leading, 30 Coincident, 9 Lagging) and 5 other time series, listed in Table 1. The composite indexes of leading, coincident, and lagging indicators produced by the Conference Board, Inc^11^. are summary statistics for the U.S. economy. The other variables are Import and Export Price Indexes, both for all commodities, Japan/U.S. Foreign Exchange Rate, M2 Money Stock, and St. Louis Adjusted Monetary Base. Data are taken from Federal Reserve (FRED) Economic Data^12^. All the time series are monthly from January 1998 to December 2017 for 20 years, 240 months in total. Description of these data is given in Supplementary Information, Section 1.

**Table 1:** Summary statistics.

No.	Acronym	Min	Max	Median	Average	Std.
**Leading indicators**						
1	AWHMAN	39.3	42.2	41.3	41.2	0.6
2	CC4WSA*	1900000.0	6540000.0	2740000.0	2996041.7	942925.1
3	NAPMNOI	23.2	71.3	55.5	55.1	7.0
4	PERMIT	513.0	2263.0	1300.0	1341.4	488.6
5	PERMIT1	337.0	1798.0	842.0	962.0	412.6
6	PERMIT5	87.0	626.0	350.0	328.4	94.6
7	USSLIND†	−2.8	2.1	1.4	1.1	0.8
8	FF	0.1	6.6	1.2	2.1	2.2
9	ACDGNO	19384.0	39864.0	33795.5	32955.6	4123.7
10	ADXTNO	109499.0	166457.0	143199.5	139864.9	14003.6
11	ACOGNO	109076.0	214933.0	166922.0	163838.9	32618.9
12	ADXDNO	131116.0	280563.0	192616.0	192164.5	24715.1
13	ACNGNO	80264.0	180908.0	134667.5	130881.2	32167.7
14	NEWORDER	46355.0	70343.0	61883.5	60410.8	6158.8
15	ANDENO	42926.0	130741.0	67443.0	67285.1	11106.8
16	NASDAQCOM	1172.1	6903.4	2462.7	2935.9	1316.4
17	T10YFFM*†	−1.2	3.7	1.8	1.6	1.3
18	STLFSI*†	−1.6	4.6	−0.4	−0.1	1.0
**Coincident indicators**						
19	USPHCI	122.3	184.2	148.3	149.5	15.6
20	PI	7373.5	16718.9	12056.0	11848.4	2670.5
21	PAYEMS	124831.0	147610.0	133111.0	134694.2	5191.0
22	TCU	66.7	84.4	77.3	77.5	3.3
23	CMRMTSPL	881646.0	1300000.0	1070000.0	1075933.4	95754.5
24	IPUTIL	82.4	108.5	99.5	98.3	5.5
25	IPMAT	78.8	110.5	93.7	94.5	8.5
26	IPBUSEQ	74.4	103.8	88.8	89.9	9.6
27	IPB51213S	86.9	125.4	103.1	103.2	10.8
28	IPB52200S	44.2	119.9	82.2	83.3	15.6
29	IPB54100S	87.5	133.7	117.4	113.3	12.1
30	IPMINE	76.9	123.1	88.7	93.5	10.8
31	IPN211111GS	68.2	122.0	82.6	89.5	13.7
32	IPN2121S	59.2	119.7	108.9	104.3	13.3
33	IPG211111CS	62.0	154.2	89.4	98.4	22.9
34	IPG211111S	66.8	142.3	83.7	93.9	20.1
35	IPG21222S	81.3	193.9	104.9	113.4	21.4
36	IPG21223S	89.8	155.9	105.7	109.5	13.8
37	IPN213111S	23.9	107.2	74.3	73.2	21.9
38	IPMAN	85.6	108.5	98.7	98.0	5.8
39	IPG321S	87.5	158.7	127.7	122.5	18.7
40	IPG332S	81.9	117.2	101.6	102.1	7.1
41	IPG3361T3S	49.4	131.7	104.7	103.1	16.1
42	IPNMAN	96.9	113.1	104.7	104.4	4.4
43	IPG3273S	87.6	181.8	140.8	132.8	25.6
44	IPG3311A2S	56.8	123.1	97.4	96.6	10.0
45	IPCONGD	97.2	114.3	104.8	105.2	4.4
46	IPDCONGD	79.4	125.9	113.6	110.8	10.5
47	IPNCONGD	98.2	112.3	102.7	103.6	3.5
48	IPFINAL	90.6	109.0	99.4	99.4	4.1
**Lagging indicators**						
49	UEMPMEAN*	12.1	40.7	19.6	23.4	8.9
50	UEMPMED*	5.2	25.2	10.0	11.6	4.9
51	MPRIME	3.2	9.5	4.2	5.2	2.1
52	BUSLOANSNSA	854.4	2124.1	1208.0	1308.5	376.8
53	CILLCBM027NBOG	409.3	1158.0	629.2	693.6	209.3
54	CILSCBM027NBOG	223.0	595.2	348.2	374.7	100.5
55	CILDCBM027NBOG	632.3	1748.1	975.2	1068.4	309.5
56	CPIAUCSL	162.0	247.9	211.4	206.5	26.1
57	INVCMRMTSPL	1220000.0	1810000.0	1490000.0	1504166.7	150786.4
**Others**						
58	IR	91.0	147.5	118.3	116.4	17.0
59	IQ	97.3	135.3	116.4	114.7	13.0
60	EXJPUS	76.6	144.7	109.5	107.9	14.5
61	M2SL*	4046.3	13833.2	7474.3	8088.3	2818.6
62	AMBSL*	512.0	4099.1	856.0	1796.0	1306.1

Minimum, maximum, median, average, and standard deviation are shown for each of the time series. Asterisk (*) indicates a variable to be inversely cycled in our analysis. Dagger (^†^) also indicates a variable which is not positive definite; the simple difference is applied to such a variable instead of the logarithmic difference.

In order to fix the direction of positive/negative growth rates in these time series to coincide with boom/bust of business cycles, the sign of several indexes such as unemployment rate is inverted as “inversely cycled variables” (indicated by asterisks); in general, unemployment rate decreases as business conditions improve. Also we remark that seasonally adjusted indexes given by the FRED Economic Data are used if available. Non-seasonally adjusted indexes are 8, 16–18, 51–55, 58–59. We verified, however, that no significant seasonality is present in all these time series (see Supplementary Figs. S1–S3).

## Methods

In order to overcome the shortcomings illustrated in the introduction, we employ novel analytical tools for identifying statistically significant correlation in the time series data, and isolate co-movements in this paper. Although many of these methods are described in the book by some of the present authors^13^, to make the present paper self-contained, we give the following concise review.

### Complex Hilbert PCA (CHPCA)

CHPCA has been successfully used in various fields, such as meteorology/climatology, signal processing, finance, and economics^13–24^. This method introduces an imaginary part to the original time series ${w}_{\alpha }(t)$, which was obtained by Hilbert transformation. We refer to the complexified signal corresponding to ${w}_{\alpha }(t)$ as ${\tilde{w}}_{\alpha }(t)$. The resulting complex correlation matrix $\tilde{{\boldsymbol{C}}}=({\tilde{C}}_{\alpha \beta })$ from $\{{\tilde{w}}_{\alpha }(t)\}$ and its eigenmodes contain information on correlations with time lead/lag: The absolute value of the complex correlation coefficient $|{\tilde{C}}_{\alpha \beta }|$ gives the strength of the correlation, whereas its phase $arg({\tilde{C}}_{\alpha \beta })$ measures to what extent the time series *β* leads the time series *α*.

### Rotational random shuffling method (RRS)

In order to identify which eigenmodes of $\tilde{{\boldsymbol{C}}}$ are the significant mode (signal) and which are the noise, we employ RRS^23,25^. In this method, we cut off the intercorrelation between the time series by randomly shuffling each of them independently and carrying out the CHPCA analysis. By doing this many times, and comparing the actual eigenvalues and the distribution of the simulated eigenvalues, we can identify which modes are significant. We then construct the significant correlation matrix ${\tilde{{\boldsymbol{C}}}}^{(\text{sig})}$ made only from significant modes and use it for the following analysis.

### Hodge decomposition

This is a tool used to untangle time lead/delay: For example, if a time series A is ${m}_{\text{AB}} > 0$ months ahead of B, which is, in turn, ${m}_{\text{B}C} > 0$ ahead of C, which is behind A by ${m}_{\text{CA}} < 0$, how should we summarize the movements of A, B, and C? In the current analysis, we have 62 time series, which make this problem difficult. The Hodge decomposition splits all the flows, namely, the phases of the significant correlation coefficients ${\tilde{C}}_{\alpha \beta }^{(\text{sig})}$, to gradient flow on one hand, and the circular flow on the other. The former is proportional to a difference in Hodge potentials of the two nodes at the ends of the link, and the latter is divergence-free flow. The resulting Hodge potential serves as a measure of hierarchical order of individual nodes.

### Clustering analysis (CA)

This method^25,26^ was inspired by percolation analysis in condensed matter physics. We define the order of items from leading to lagging by their Hodge potential. Then the variable ${w}_{\alpha }(t)$ is linked with its nearest neighbor ${w}_{\beta }(t{\prime} )$ if they are similar. This procedure creates two types of clusters; one made by combining positive changes and the other by aggregating negative changes.

### Synchronization network

One can visualize the lead/lag relationship between nodes, using the link created by the significant correlation matrix ${\tilde{{\boldsymbol{C}}}}^{(\text{sig})}$, utilizing the Hodge potential.

Details of the above methods are given in Supplementary Information, Section 3.

## Results

### Eigenvalue distribution

Among all the eigenmodes of the complex correlation matrix $\tilde{{\boldsymbol{C}}}$, we have found that those with the six largest eigenvalues of the complex correlation matrix $\tilde{{\boldsymbol{C}}}$ are statistically significant from the RRS analysis. The largest eigenvalue ${\tilde{\lambda }}_{1}=14.18$ satisfies 22.9% of the exact sum rule, ${\sum }_{\ell \mathrm{=1}}^{N}{\tilde{\lambda }}_{\ell }=N$, and the accumulation of the six significant eigenvalues, 55.4% of the sum rule. We thus see that about half of the total strength of fluctuations in the macro indicators can be explained just by random noise. Details are given in the Supplementary Information, Section 4.1.

### Significant eigenvectors

**Figure 1:**
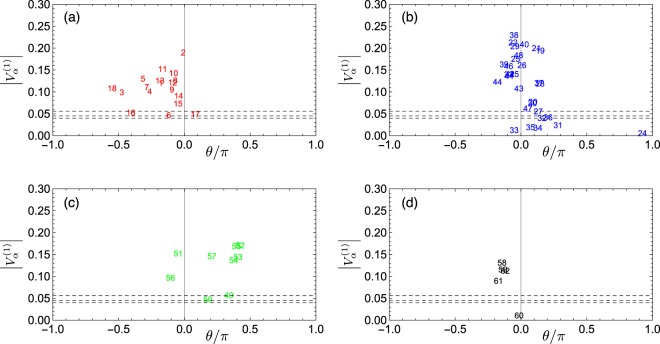
Lead-lag relationship among the macro variables represented by the most dominant eigenvector. The panels (a–d) show the results for the leading, coincident, lagging, and other macro indicators, respectively; the average over all phases is reset to be zero as indicated by the gray vertical lines. The absolute values of individual components in the eigenvector are plotted against their phases in each panel. The three dotted horizontal lines are criteria to identify significant components with 1, 5, and 10% significance levels, aligned from top to bottom.

The components of the first eigenvector are shown in Fig. 1 on the phase-absolute value plane. The larger the absolute value, the more important its role in the comovement. The statistical significance level is determined by adding a random time series as the 63rd component and by measuring its magnitude. We have carried out this simulation 10^4^ times and determined the 1, 5 and 10% significance levels shown in this plot, making sure that the correlation structure of the original data (less the random time series) is kept unchanged.

In Fig. 1, time runs from left to right; that is, components are ahead of those located on their right-hand side. On the whole, these results confirm the assignment of leading, coincident, and lagging to each of the macro indicators by the Conference Board. We, however, find that some indicators are categorized incorrectly. To be specific, the two lagging indicators, Bank Prime Loan Rate (#51) and Consumer Price Index for All Urban Consumers: All Items (#56), appear to be regarded as coincident indicators. Furthermore, all of the other macro indicators, excluding Japan / U.S. Foreign Exchange Rate (#60), move coherently as front runners in the coincident indicators or as rear runners in the leading indicators. We elaborate on this issue in later subsections.

Further details of the significant eigenvectors are discussed in Supplementary Information, Section 4.2.

### Comparison with the PCA

To demonstrate the advantages of the CHPCA over the ordinary PCA, we repeated the same calculations as in the previous subsections, but within PCA, and compared the results with the corresponding results of CHPCA. The PCA totally depends on a real correlation coefficient matrix at equal time. The eigenvalues for the correlation matrix are positive definite as well as those of CHPCA. On the other hand, components of the eigenvectors are real while those of CHPCA are complex.

The parallel analysis based on the RRS tells us that the eight largest eigenvalues of the PCA are significant; there are two extras, in contrast to the CHPCA.

A similarity measure has been defined to make an explicit connection between the eigenvectors of CHPCA and those of PCA. Using the similarity measure we found that each aspect (real or imaginary) of the eigenvectors of CHPCA has a corresponding eigenvector of PCA. For instance, the real part of the first eigenvector of CHPCA is virtually indistinguishable from the corresponding eigenvector of PCA. Much more importantly, the imaginary part of the first eigenvector of CHPCA resembles the second and third eigenvectors of PCA. We thus see that the two orthogonal aspects of the first eigenvector are well described by the three eigenvectors of PCA. Certainly, partial information on the dynamic correlations between macroeconomic indicators is carried by the three eigenvectors of PCA. However, one could hardly reach the whole picture on the comovement of indicators as simply manifested in the first eigenvector of CHPCA with the results of PCA alone; reconstruction of the first eigenvector of CHPCA out of the three eigenvectors of PCA is very difficult.

These results allow us to conclude that the CHPCA is much better than the PCA for investigation of dynamic correlations involved in complex systems, including economic cycles. We refer the readers to the Supplementary Information, Section 4.3 for details of the comparison between CHPCA and PCA.

### Hodge synchronization network

In order to address the issue noted above, namely the leading/coincidental/lagging property of the indicators, we need to take into account all six eigenmodes. This can be readily done by examining the significant correlation matrix made of only the significant eigenvectors (see (S12)).

The absolute value $|{\tilde{C}}_{\alpha 63}^{(\text{sig})}|$ is the measure of the strength of the correlation between the indicator *α* and *β*. However, due to the fact that the time-range is finite, it is not equal to zero even if there is no correlation. In order to remove those fictitious correlations, we have introduced a random time series as the 63rd data and calculated $|{\tilde{C}}_{\alpha 63}^{(\text{sig})}|$ for $\alpha =\mathrm{1,2,}\cdots 62$. Having done this 10,000 times, we have found that fictitious correlation can be excluded by the criteria $|{\tilde{C}}_{\alpha \beta }^{(\text{sig})}| > 0.195$ with 5% errors.

The phase of the remaining matrix elements is worth studying for the time-structure, as the phase ${\tilde{C}}_{\alpha \beta }^{(\text{sig})}$ corresponds to the lead time of the indicator *β* over the indicator *α*. If, however, ${\tilde{C}}_{\alpha \beta }^{(\text{sig})}=-\,1$, this means that they are anti-correlated. Therefore, for $\text{Re}[{\tilde{C}}_{\alpha \beta }^{(\text{sig})}] < 0$, we determine the indicator *α* and *β* to be anti-correlated with time lead determined by its phase minus $\pi$.

In this manner, we obtain a network, where links are made of the remaining ${\tilde{C}}_{\alpha \beta }^{(\text{sig})}$ and flows are the phases determined above. We name this **Hodge Synchronization Network** (HSN).

**Figure 2:**
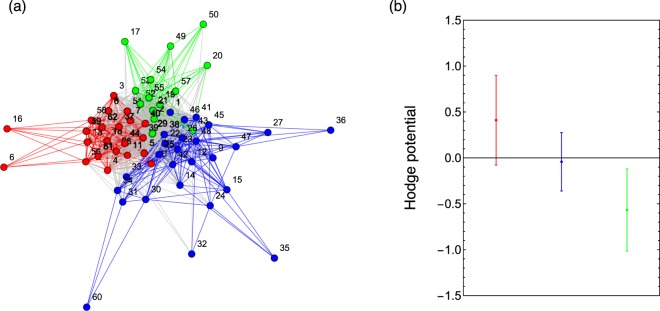
Panel (a): Community decomposition of Hodge Synchronization Network, where the nodes in the same community are shown in same color. Panel (b): Average values and standard deviation of the Hodge potentials of the three communities shown in the panel (a), where red community is leading, blue is coincidental, and green lagging.

Community decomposition of HSN leads to three communities, as seen in the left panel of Fig. 2. Here we detected the communities by maximizing the modularity with the greedy algorithm, and obtained the optimized layout for the network in a spring-electrical model.

We carry out the Hodge decomposition of this network. This is because the Hodge Potential is made of flows: The smaller the flow, the smaller the difference of the Hodge potentials of the pair of nodes, and vice versa. This property guarantees that the Hodge potential of each indicator reflects its lead time. The right panel of Fig. 2 shows the average and standard deviation of the Hodge potentials of each community. We find here that the red community is leading, the blue coincidental, the green lagging.

**Figure 3:**
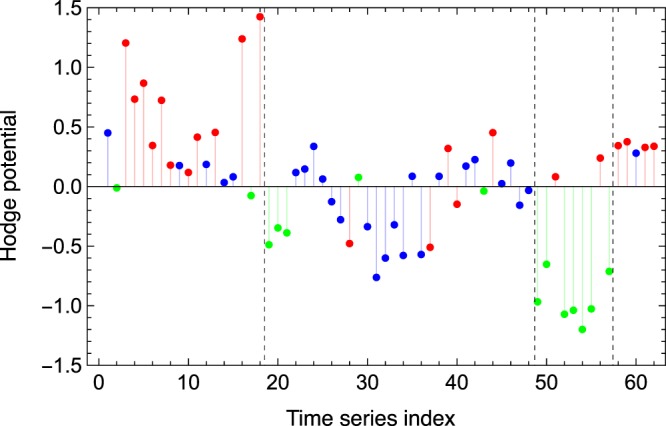
Categorization of the indicators. The vertical dashed lines divide the indicators to Leading, Coincidental, Lagging and others from left to right. The color coding for the indicators is the same as in Fig. 2.

The Hodge potential values for individual indicators in each of the original classifications are given in Fig. 3, where the indicators are also classified according to which community they belong to. Here we observe that the indicators’ categorization by community is almost identical to the one by the original assignment. This result opens a door for realizing the algorithmic categorization of a given data set of macroeconomic time series.

**Figure 4:**
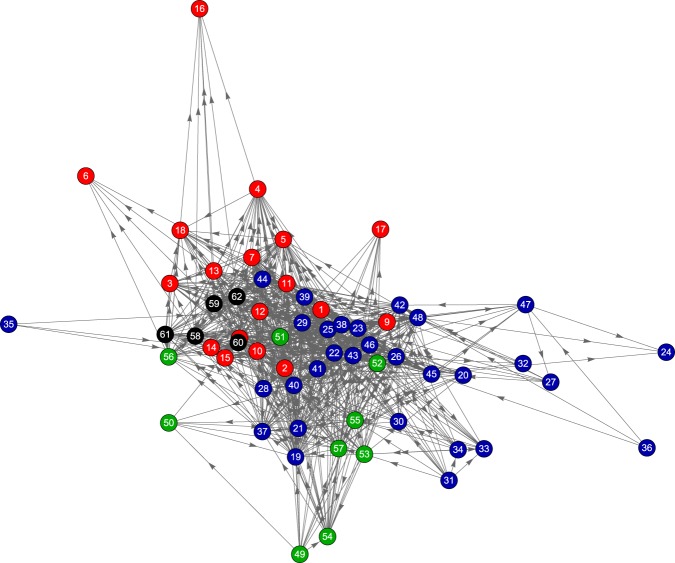
The Hodge Synchronization Network, where the vertical coordinate is the Hodge potential.

Using the value of the Hodge potentials as the vertical coordinate and determining the horizontal coordinate by the Charge-Spring algorithm, we obtain the synchronization network shown in Fig. 4. Due to the construction, the indicators are listed from top to bottom in the leading order determined by their Hodge potentials.

### Mode signals

**Figure 5:**
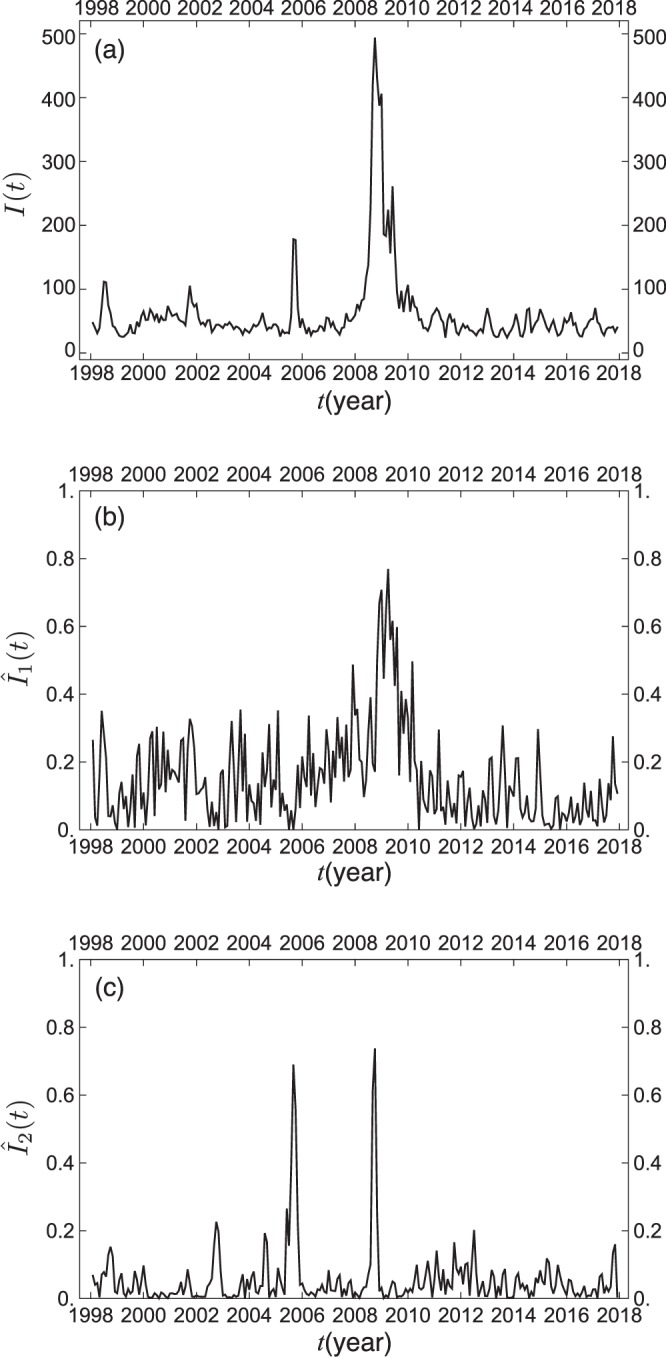
Temporal variation of (**a**) the total intensity of fluctuations in the macro indicators, (**b**) the relative mode intensity for the first eigenmode, and (**c**) the relative mode intensity for the second eigenmode.

Figure 5(a) demonstrates the volatility of the U.S. economy by calculating the total intensity $I(t)={\sum }_{\alpha \mathrm{=1}}^{N}|{\tilde{w}}_{\alpha }(t{)|}^{2}$ of fluctuations in the macro indicators. The main peak consisting of several spikes almost spans the period of the subprime mortgage crisis. Also one can observe three sharp peaks around the middle of 1998, the end of 2001, and the summer of 2005, ahead of the main peak.

Each of the complexified time series, to which the standardization procedure has been applied, is expanded in terms of the eigenvectors $\{{\tilde{{\boldsymbol{V}}}}^{(\ell )}\}$ of CHPCA:

1 $$\tilde{{\boldsymbol{w}}}(t)=\mathop{\sum }\limits_{\alpha =1}^{N}{\tilde{w}}_{\alpha }(t){{\boldsymbol{e}}}_{\alpha }=\mathop{\sum }\limits_{\ell \mathrm{=1}}^{N}{a}_{\ell }(t){\tilde{{\boldsymbol{V}}}}^{(\ell )},$$

where ${{\boldsymbol{e}}}_{\alpha }$ is the basis vector of the *α*-th component, e.g., the transpose of ${{\boldsymbol{e}}}_{1}$ is given by ${{\boldsymbol{e}}}_{1}^{t}\mathrm{=(1,0,}\cdots \mathrm{,0)}$. The coefficient ${a}_{\ell }(t)$ is referred to as *mode signal* of the $\ell$-th eigenmode. The relative mode intensity ${\hat{I}}_{\ell }(t)$ is defined by the following:

2 $${\hat{I}}_{\ell }(t)=\frac{{I}_{\ell }(t)}{I(t)}=\frac{|{a}_{\ell }(t{)|}^{2}}{\mathop{\sum }\limits_{\ell \mathrm{=1}}^{N}|{a}_{\ell }(t{)|}^{2}},\,{I}_{\ell }(t)=|{a}_{\ell }(t{)|}^{2}\mathrm{}.$$

which calculates the fractional contribution of each eigenmode to the overall strength of indicator fluctuations at every instant of time.

Figure 5(b,c) display the relative intensities, ${\hat{I}}_{1}(t)$ and ${\hat{I}}_{2}(t)$, of the mode signals of the two dominant eigenmodes as a function of time. The three major peaks except for the second largest peak in the total volatility $I(t)$ as shown in Fig. 5(a) are explained by the first eigenmode. In contrast, the second largest peak in the middle of 2005 is almost entirely ascribed to the second eigenmode. Also the leading subpeak, constituting the main peak of $I(t)$, coincides with the largest peak of ${\hat{I}}_{2}(t)$ located in the summer of 2008. We recall that Hurricane Katrina hit the Gulf area in August 2005 and the oil bubble burst in July 2008. Needless to say, these events had profound influences on the oil and oil-related industries in the U.S. Certainly, the second eigenmode is well demonstrated by the industrial production indexes for crude petroleum and natural gas extraction (#34), crude oil (#33), mining (#30), and mining of natural gas (#31), as shown by Fig. S5 in Supplementary Information. We thus see that the total intensity of the macroeconomic fluctuations is clearly resolved by the two dominant eigenmodes, with well-defined economic interpretations.

### Economic cycles

**Figure 6:**
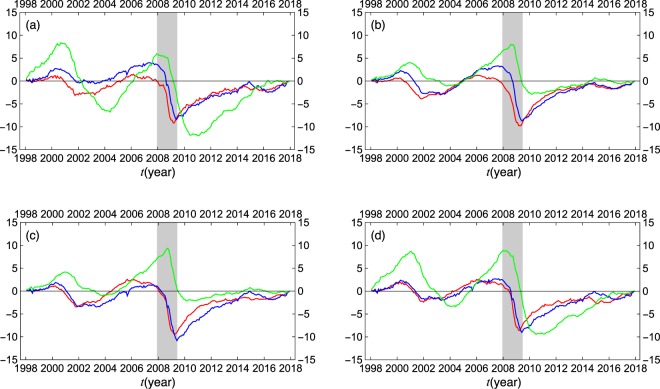
Comparison of the representative leading (red), coincident (blue), and lagging (green) indexes with the equivalent indexes described by the dominant eigenmodes alone. The panel (a) shows temporal accumulation of the standardized log-difference or simple difference of the original data averaged over the leading, coincident, and lagging categories. The panels (b–d) show the corresponding results obtained by selecting the first eigenmode alone, the first and second eigenmodes, and all of the six significant eigenmodes, respectively. The shaded area indicates the recession phase (December 2007 – May 2009) due to the Lehman crisis.

The mode signals ${a}_{\ell }(t)$’s enable us to see to what extent economic cycles are described by the significant eigenmodes. We first construct representative leading, coincident, and lagging indexes by averaging the standardized log-difference or simple difference of the original data over each of the three categories. The results are given in the panel (a) of Fig. 6, where the representative indexes are successively accumulated in the time direction. The panels (b), (c), and (d) of Fig. 6 compare the results based on the original data with the corresponding results obtained by selecting the first eigenmode alone, the first and second eigenmodes, and all of the six significant eigenmodes, respectively.

The essential features of the economic cycles, including the Great Recession after the Lehman crisis, are well explained by the first eigenmode alone. However, we see that the strength of the cyclic behavior of the lagging indicator is underestimated in the first eigenmode. The quantitative agreement with the original data is progressively improved by taking account of the contribution of the significant eigenmodes term by term, from the first mode up to the sixth mode. The results obtained with full account of the significant eigenmodes are almost indistinguishable from those based on the original data.

### Cluster analysis

**Figure 7:**
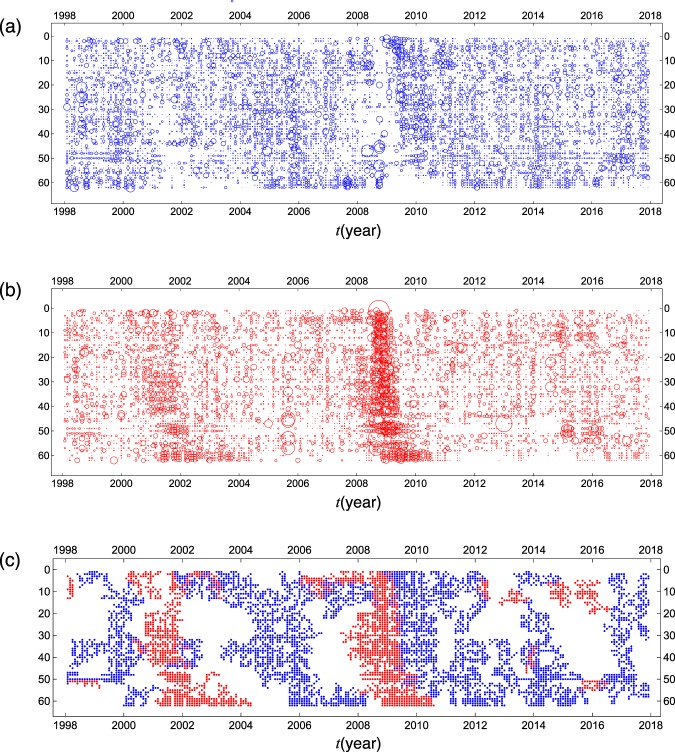
Standardized monthly log differences of all the macro indicators are visualized together with the results of the cluster detection in a matrix representation. The magnitude of positive and negative changes of the indicators is depicted by circles in the panels (a,b), separately. Time passes from left to right in the horizontal direction. The variables are ordered according to their Hodge potentials from top (leading) to bottom (lagging) in the vertical direction. The panel (c) shows the 20 largest clusters detected by the percolation analysis with ${g}_{c}=0.45$ and 0.6 for positive and negative changes of the macro indicators, respectively, as displayed in the panels (a,b).

Propagation of macroeconomic shocks across the individual indexes from leading to lagging are displayed in Fig. 7(a,b), where the indicators are ordered vertically from top (leading) to bottom (lagging) not by their original identification numbers (1–62) but by their Hodge potentials, as shown in Fig. 3.

Although we can visually observe clusters of the indicators, it remains only subjective. As has been described in the previous section, we adopt the percolation model to identify clusters in an algorithmic way. Obviously, clusters thus detected depend crucially on the choice of the threshold *g*_*c*_. If we adopt a too small value of *g*_*c*_, the indicators would fragment to a number of tiny pieces. For a too large value of *g*_*c*_, on the other hand, most of the indicators would be connected to form a single group. If we carefully adjust *g*_*c*_ close to the percolation threshold in the indicator lattice system, various scales of clusters are formed with a power law distribution. Near the percolation threshold, we can thereby extract information on the clustering properties of indicators in the most effective way. This algorithm for detecting clusters is illustrated in Supplementary Fig. S4. By reiterating the percolation calculations with varied *g*_*c*_, we have found that a percolation transition takes place around *g*_*c*_ = 0.45 and 0.6 in the model system for positive and negative changes of the indicators, respectively. Incidentally, the total numbers of clusters thus obtained are 306 and 479 for positive and negative changes of the indicators. The results are shown in Fig. 7(c). The shock propagations due to the dot-com crash in March 2000 and the subprime mortgage crisis starting from December 2007 are detected as two large clusters of negative changes in the indicators. Also the recoveries from those severe recessions form two large clusters of positive changes in the indicators.

## Discussion

Understanding the challenges ahead of us, we suggest that the roles of macroeconomic indicators might change with time and with fluctuating economic dynamics, and real-time analysis using noise-reducing methodologies might be appropriate to offer improved forecasting of the business cycle.

In this paper we study 57 US macroeconomic indicators as well as 5 money/trade indexes and their relationships with the US business cycle. We analyze the importance of various macroeconomic indicators and their leading/lagging roles with respect to economic expansions and contractions. We build on almost a century’s worth of research in investigating dynamic societal processes, such as innovation, and the influence of such processes on economic behavior. Business cycles reflect trends of economic activities captured by specific macroeconomic indicators whose characteristics we examine in this study.

We expand the set of methodological approaches to analyzing business cycles and important economic turning points by proposing novel methods such as the Hodge Decomposition for hierarchically ordering macroeconomic indicators by their leading/lagging roles in relation to business cycles. Additionally, we extract significant information by using noise-reducing algorithms to identify economically significant events. Moreover, by using a synchronization network approach and clustering analysis of temporal positive and negative changes of macroeconomic indicators, we find significant consecutive collective behavior among macroeconomic indicators.

In our study, first we addressed the question of identifying the most prominent leading macroeconomic indicators within the 20-year time period that we analyze, between Jan. 1998 and Dec. 2017. We found that, besides the indicators classified as leading by the Conference Board, some of the coincident and lagging indicators as well as certain money/trade indexes show lead indicator characteristics. Namely, coincidental indicators such as the industrial production indexes for wood product manufacturing (#39) and for iron and steel manufacturing (#44) seem to be indicative of the business cycle. Similarly, the lagging indicators: Bank Prime Loan Rate (#51) and the Consumer Price Index (#56) surface as lead business cycle indicators. Additionally, the import and export price indexes (#58) and (#59) respectively, as well as the M2-Money Stock (#61) and the adjusted monetary base (#62) seem to precede turning points in the business cycle.

Second, we have used our CHPCA and Hodge decomposition methodologies (described in detail in the methods section) to identify specific, significant economic events by analyzing the fluctuation intensity in macroeconomic indexes. We were able to identify the impact of the Global Financial Crisis of 2007–2009 by the largest eigenvalue mode signal, extract the devastating effect of Hurricane Katrina in August 2005, and point out the Oil Price Crisis of July 2008 by analyzing the mode signal of the second largest eigenvector, using the CHPCA approach.

Third, we classified the macroeconomic indicators hierarchically by their leading positions, as obtained by our methodologies, and then performed cluster analysis, defining clusters as collective consecutive positive or negative changes in the indicators. We found that the largest negative clusters in the leading indicator time series occur between 2000–2002, with the lagging indicators showing clusters of negative changes up to 2004. Another prominent negative change cluster appears as early as 2006 in the leading indicators and persists until 2011 in the lagging indicators. In addition to these two significant negative clusters, two large positive clusters appear, one between 2004 and 2006 with some indicators showing positive changes up to 2008, and another between 2009 and 2012.

Our results show that most of the variability in the macroeconomic indicators can be explained by only six significant eigenvectors, out of the 62 time series analyzed. We can interpret this result as having a small number of significant sources of macroeconomic variability. There are several possible directions for future research based on the results and the issues that we faced in this study. First, we used monthly data, and changing the data resolution to either weekly or daily may improve the findings. Second, we conducted the analysis only for U.S. macroeconomic indicators, and a future research direction may include application of our proposed methodologies to more countries and even regions or unions such as the European Union. Third, we based our investigations on only 62 time series covering a 20-year period, and if we increase the data set or complement it by including pricing data, the results may improve. Finally, creating a comprehensive dynamic forecasting tool of business cycles requires continuous, ongoing further research. One such future research direction might include improving the proposed methodology in this paper or taking another methodological approach including a different data set in the future.

## Supplementary information

Supplementary information.
