# Publisher Correction: Relationship between Macroeconomic Indicators and Economic Cycles in U.S.

**DOI:** 10.1038/s41598-020-70100-3

**Published:** 2020-08-04

**Authors:** Hiroshi Iyetomi, Hideaki Aoyama, Yoshi Fujiwara, Wataru Souma, Irena Vodenska, Hiroshi Yoshikawa

**Affiliations:** 1 Department of Mathematics, Niigata University, Niigata, 950-2181 Japan; 2 Kyoto University, GSAIS, Kyoto, 606-8502 Japan; 3 Research Institute of Economy, Trade and Industry (RIETI), Tokyo, 100-8901 Japan; 4 Graduate School of Simulation Studies, University of Hyogo, Kobe, 650-0047 Japan; 5 College of Science and Technology, Nihon University, Funabashi, 274-8501 Japan; 6 Metropolitan College, Boston University, Boston, MA 02215 USA; 7 Faculty of Economics, Rissho University, Tokyo, 141-8602 Japan

Correction to: *Scientific Reports*https://doi.org/10.1038/s41598-020-65002-3, published online 21 May 2020

The original version of this Article contained an error in the title of the paper, where “U.S.” was incorrectly given as “U.S”.

Additionally, there was a repeated typographical error where ‘coincidental’

now reads:

‘coincident’.

These errors have now been corrected in the PDF and HTML versions of the Article.

