# Supplementary material for: Relationship between Macroeconomic Indicators and Economic Cycles in U.S
Source: Sci Rep. 2020 May 21;10:8420. doi: 10.1038/s41598-020-65002-3 (PMC7242465; doi:10.1038/s41598-020-65002-3)
Supplement: Supplementary file 1 — Supplementary information. [file 41598_2020_65002_MOESM1_ESM.pdf]

Relationship between Macroeconomic Indicators and Economic Cycles in U.S.

– Supplementary Information

Hiroshi Iyetomi, Hideaki Aoyama, Yoshi Fujiwara,
Wataru Souma, Irena Vodenska and Hiroshi Yoshikawa

1 Data description

In the main body of the paper, only abbreviated names were used for macroeconomic indicators. Their full name and the descriptions are given below. For complete information, refer to web page of Federal Reserve (FRED) Economic Data[1] and others, indicated after each acronym of index.

1. AWHMAN `https://fred.stlouisfed.org/series/AWHMAN`
   Average weekly hours relate to the average hours per worker for which pay was received and is different from standard or scheduled hours. Factors such as unpaid absenteeism, labor turnover, part-time work, and stoppages cause average weekly hours to be lower than scheduled hours of work for an establishment. Group averages further reflect changes in the workweek of component industries. Average weekly hours are the total weekly hours divided by the employees paid for those hours.

   Production and related employees include working supervisors and all nonsupervisory employees (including group leaders and trainees) engaged in fabricating, processing, assembling, inspecting, receiving, storing, handling, packing, warehousing, shipping, trucking, hauling, maintenance, repair, janitorial, guard services, product development, auxiliary production for plant's own use (for example, power plant), record-keeping, and other services closely associated with the above production operations.

   Nonsupervisory employees include those individuals in private, service-providing industries who are not above the working-supervisor level. This group includes individuals such as office and clerical workers, repairers, salespersons, operators, drivers, physicians, lawyers, accountants, nurses, social workers, research aides, teachers, drafters, photographers, beauticians, musicians, restaurant workers, custodial workers, attendants, line installers and repairers, laborers, janitors, guards, and other employees at similar occupational levels whose services are closely associated with those of the employees listed.

   The series comes from the 'Current Employment Statistics (Establishment Survey).'

   The source code is: CES3000000007

2. CC4WSA `https://fred.stlouisfed.org/series/CC4WSA`
   Insured unemployment is the number of people receiving unemployment benefits.

3. NAPMNOI `https://ycharts.com/indicators/new_orders_index`
   Compiled by the Institute for Supply Management (ISM), this report is one of the first released each month that has a major impact on the markets. Based on a survey of purchasing managers, it reports information about purchase orders, production, employment, and more in the manufacturing industry.

4. PERMIT `https://fred.stlouisfed.org/series/PERMIT`
   Starting with the 2005-02-16 release, the series reflects an increase in the universe of permit-issuing places from 19,000 to 20,000 places.

5. PERMIT1 `https://fred.stlouisfed.org/series/PERMIT`
   Starting with the 2005-02-16 release, the series reflects an increase in the universe of permit-issuing places from 19,000 to 20,000 places.

6. PERMIT5 https://fred.stlouisfed.org/series/PERMIT5
Starting with the 2005-02-16 release, the series reflects an increase in the universe of permit-issuing places from 19,000 to 20,000 places.

7. USSLIND https://fred.stlouisfed.org/series/USSLIND
The leading index for each state predicts the six-month growth rate of the state's coincident index. In addition to the coincident index, the models include other variables that lead the economy: state-level housing permits (1 to 4 units), state initial unemployment insurance claims, delivery times from the Institute for Supply Management (ISM) manufacturing survey, and the interest rate spread between the 10-year Treasury bond and the 3-month Treasury bill.

8. FF https://fred.stlouisfed.org/series/FF
The federal funds rate is the interest rate at which depository institutions trade federal funds (balances held at Federal Reserve Banks) with each other overnight. When a depository institution has surplus balances in its reserve account, it lends to other banks in need of larger balances. In simpler terms, a bank with excess cash, which is often referred to as liquidity, will lend to another bank that needs to quickly raise liquidity. (1) The rate that the borrowing institution pays to the lending institution is determined between the two banks; the weighted average rate for all of these types of negotiations is called the effective federal funds rate.(2) The effective federal funds rate is essentially determined by the market but is influenced by the Federal Reserve through open market operations to reach the federal funds rate target.(2) The Federal Open Market Committee (FOMC) meets eight times a year to determine the federal funds target rate. As previously stated, this rate influences the effective federal funds rate through open market operations or by buying and selling of government bonds (government debt).(2) More specifically, the Federal Reserve decreases liquidity by selling government bonds, thereby raising the federal funds rate because banks have less liquidity to trade with other banks. Similarly, the Federal Reserve can increase liquidity by buying government bonds, decreasing the federal funds rate because banks have excess liquidity for trade. Whether the Federal Reserve wants to buy or sell bonds depends on the state of the economy. If the FOMC believes the economy is growing too fast and inflation pressures are inconsistent with the dual mandate of the Federal Reserve, the Committee may set a higher federal funds rate target to temper economic activity. In the opposing scenario, the FOMC may set a lower federal funds rate target to spur greater economic activity. Therefore, the FOMC must observe the current state of the economy to determine the best course of monetary policy that will maximize economic growth while adhering to the dual mandate set forth by Congress. In making its monetary policy decisions, the FOMC considers a wealth of economic data, such as: trends in prices and wages, employment, consumer spending and income, business investments, and foreign exchange markets. The federal funds rate is the central interest rate in the U.S. financial market. It influences other interest rates such as the prime rate, which is the rate banks charge their customers with higher credit ratings. Additionally, the federal funds rate indirectly influences longer- term interest rates such as mortgages, loans, and savings, all of which are very important to consumer wealth and confidence.(2)
References
(1) Federal Reserve Bank of New York. "Federal funds." Fedpoints, August 2007.
(2) Board of Governors of the Federal Reserve System. "Monetary Policy". http://www.federalreserve.gov/monetarypolicy/default.htm.

9. ACDGNO https://fred.stlouisfed.org/series/ACDGNO
This series is a topical regrouping of the separate industry categories.

10. ADXTNO https://fred.stlouisfed.org/series/ADXTNO
This series is a topical regrouping of the separate industry categories.

11. ACOGNO https://fred.stlouisfed.org/series/ACOGNO
This series is a topical regrouping of the separate industry categories.

12. ADXDNO https://fred.stlouisfed.org/series/ADXDNO
This series is a topical regrouping of the separate industry categories.

13. ACNGNO https://fred.stlouisfed.org/series/ACNGNO
This series is a topical regrouping of the separate industry categories.

    This series is identical to the Value of Manufacturers' Shipments for Consumer Goods: Consumer Nondurable Goods Industries https://fred.stlouisfed.org/series/ACNGVS and is also presented on Table 5 at https://www.census.gov/manufacturing/m3/prel/pdf/s-i-o.pdf.

14. NEWORDER https://fred.stlouisfed.org/series/NEWORDER
Effective May 21, 2001, data were reconstructed to reflect the switch from the Standard Industrial Classification (SIC) system to the North American Industry Classification System (NAICS).

15. ANDENO https://fred.stlouisfed.org/series/ANDENO
This series is a topical regrouping of the separate industry categories. Nondefense capital goods industries include: small arms and ordnance; farm machinery and equipment; construction machinery; mining, oil, and gas field machinery; industrial machiner.

16. NASDAQCOM https://fred.stlouisfed.org/series/NASDAQCOM
The NASDAQ Composite Index is a market capitalization weighted index with more than 3000 common equities listed on the NASDAQ Stock Market. The types of securities in the index include American depositary receipts (ADRs), common stocks, real estate investment trusts (REITs), and tracking stocks. The index includes all NASDAQ listed stocks that are not derivatives, preferred shares, funds, exchange-traded funds (ETFs) or debentures. Copyright ©2016, NASDAQ OMX Group, Inc.

17. T10YFFM https://fred.stlouisfed.org/series/T10YFFM
Series is calculated as the spread between 10-Year Treasury Constant Maturity (https://fred.stlouisfed.org/series/GS10) and Effective Federal Funds Rate (https://fred.stlouisfed.org/series/FEDFUNDS).

18. STLFSI https://fred.stlouisfed.org/series/STLFSI
The STLFSI measures the degree of financial stress in the markets and is constructed from 18 weekly data series: seven interest rate series, six yield spreads and five other indicators. Each of these variables captures some aspect of financial stress. Accordingly, as the level of financial stress in the economy changes, the data series are likely to move together.

    How to Interpret the Index: The average value of the index, which begins in late 1993, is designed to be zero. Thus, zero is viewed as representing normal financial market conditions. Values below zero suggest below-average financial market stress, while values above zero suggest above-average financial market stress.

    More information: For additional information on the STLFSI and its construction, see "Measuring Financial Market Stress" (https://files.stlouisfed.org/research/publications/es/10/ES1002.pdf) and the related appendix (https://files.stlouisfed.org/files/htdocs/publications/net/NETJan2010Appendix.pdf).

    For a list of the components that are used to construct the STLFSI see https://www.stlouisfed.org/news-releases/st-louis-fed-financial-stress-index/stlfsi-key.

    As of 07/15/2010 the Vanguard Financial Exchange-Traded Fund series has been replaced with the S&P 500 Financials Index. This change was made to facilitate a more timely and automated updating of the FSI. Switching from the Vanguard series to the S&P series produced no meaningful change in the index.

    Copyright, 2016, Federal Reserve Bank of St. Louis.

19. USPHCI https://fred.stlouisfed.org/series/USPHCI
The Coincident Economic Activity Index includes four indicators: nonfarm payroll em

20. PI https://fred.stlouisfed.org/series/PI
BEA Account Code: A065RC Personal income is the income that persons receive in return for their provision of labor, land, and capital used in current production and the net current transfer payments that they receive from business and from government.25 Personal income is equal to national income minus corporate profits with inventory valuation and capital consumption adjustments, taxes on production and imports less subsidies, contributions for government social insurance, net interest and miscellaneous payments on assets, business current transfer payments (net), current surplus of government enterprises, and wage accruals less disbursements, plus personal income receipts on assets and personal current transfer receipts. A Guide to the National Income and Product Accounts of the United States (NIPA) - (http://www.bea.gov/national/pdf/nipaguid.pdf)

21. PAYEMS https://fred.stlouisfed.org/series/PAYEMS
All Employees: Total Nonfarm, commonly known as Total Nonfarm Payroll, is a measure of the number of U.S. workers in the economy that excludes proprietors, private household employees, unpaid volunteers, farm employees, and the unincorporated self-employed. This measure accounts for approximately 80 percent of the workers who contribute to Gross Domestic Product (GDP).

This measure provides useful insights into the current economic situation because it can represent the number of jobs added or lost in an economy. Increases in employment might indicate that businesses are hiring which might also suggest that businesses are growing. Additionally, those who are newly employed have increased their personal incomes, which means (all else constant) th

Generally, the U.S. labor force and levels of employment and unemployment are subject to fluctuations due to seasonal changes in weather, major holidays, and the opening and closing of schools. The Bureau of Labor Statistics (BLS) adjusts the data to offset the seasonal effects to show non-seasonal changes: for example, women's participation in the labor force; or a general decline in the number of employees, a possible indication of a downturn in the economy. To closely examine seasonal and non-seasonal changes, the BLS releases two monthly statistical measures: the seasonally adjusted All Employees: Total Nonfarm (PAYEMS) and All Employees: Total Nonfarm (PAYNSA), which is not seasonally adjusted.

The series comes from the 'Current Employment Statistics (Establishment Survey).'

The source code is: CES0000000001

22. TCU https://fred.stlouisfed.org/series/TCU
Capacity Utilization: Total Industry (TCU) is the percentage of resources used by corporations and factories to produce goods in manufacturing, mining, and electric and gas utilities for all facilities located in the United States (excluding those in U.S. territories).(1) We can also think of capacity utilization as how much capacity is being used from the total available capacity to produce demanded finished products.

    Capacity utilization indexes are constructed for 71 industries in manufacturing, 16 in mining, and 2 in utilities. (1) Physical data on capacity utilization are primarily compiled from trade sources and government sources, such as the U.S. Geological Survey and the U.S. Energy Information Administration.(1) When physical data are unavailable, capacity utilization data are compiled from the U.S. Census Bureau's Quarterly Survey of Plant Capacity Utilization, which provides data for almost 70 percent of total industry capacity.(1) Additionally, the capacity index is developed on a monthly basis, designed to be consistent with the production index.(1) According to the Board of Governors of the Federal Reserve System, the capacity index tries to conceptualize the idea of sustainable maximum output, which is defined as the highest level of output a plant can sustain within the confines of its resources. The Board of Governors defines the seasonally adjusted capacity utilization rate as the output index divided by the capacity index. The capacity utilization rate can also implicitly describe how efficiently the factors of production (inputs in the production process) are being used. (1) It sheds light on how much more firms can produce without additional costs. Additionally, this rate gives manufacturers some idea as to how much consumer demand they will be able to meet in the future. The Federal Reserve strives to construct a capacity index consistent with time by using different relevant data sources.(1) Developing an index that is reasonable given the time period is the primary aim for this index, but there are still some difficulties. Extensive technological and structural changes have and will continue to occur, affecting the degree of tightness the Federal Reserve index of capacity utilization will represent.(2) In addition, each series of capacity utilization is flawed by commission; therefore, they should be used with caution.(2)

    References
(1) Board of Governors of the Federal Reserve System. "Industrial Production and Capacity Utilization." Statistical release G.17;. May 15, 2013. (2) Bauer, Paul W. and Deily, Mary E. "A User's Guide to Capacity–Utilization Measures." Economic Commentary. Federal Reserve Bank of Cleveland, July 1, 1988; https://www.clevelandfed.org/newsroom-and-events/publications/economic-commentary/economic-commentary-archives/1988-economic-commentaries/ec-19880701-a-users-guide-to-capacity-utilization-measures.aspx.

23. CMRMTSPL https://fred.stlouisfed.org/series/CMRMTSPL
Real Manufacturing and Trade Industries Sales (CMRMTSPL) was first constructed by the Federal Reserve Bank of St. Louis in June 2013. It is calculated using Real Manufacturing and Trade Industries Sales (HMRMT) (https://fred.stlouisfed.org/series/HMRMT) and Real Manufacturing and Trade Industries (CMRMT) (https://fred.stlouisfed.org/series/CMRMT).

    Before January 1997

    lag1(HMRMT) = one observation earlier than current time period observation

    HMRMT_PC = the growth rate of HMRMT

    lead1(CMRMTSPL) = one observation later than current time period observation

HMRMT_PC = [HMRMT/lag1(HMRMT)-1]

CMRMTSPL= lead1(CMRMTSPL)/(1+HMRMT_PC)

After December 1996

CMRMTSPL= CMRMT

24. IPUTIL https://fred.stlouisfed.org/series/IPUTIL
Industrial Production - Electric and Gas Utilities

25. IPMAT https://fred.stlouisfed.org/series/IPMAT
Industrial Production - Materials

26. IPBUSEQ https://fred.stlouisfed.org/series/IPBUSEQ
Industrial Production - Business Equipment

27. IPB51213S https://fred.stlouisfed.org/series/IPB51213S
The industrial production (IP) index measures the real output of all relevant establishments located in the United States, regardless of their ownership, but not those located in U.S. territories. (Industrial Production: Nondurable Goods: Chemical products)

28. IPB52200S https://fred.stlouisfed.org/series/IPB52200S
The industrial production (IP) index measures the real output of all relevant establishments located in the United States, regardless of their ownership, but not those located in U.S. territories. (Industrial Production: Oil and gas well drilling and manufactured homes)

29. IPB54100S https://fred.stlouisfed.org/series/IPB54100S
The industrial production (IP) index measures the real output of all relevant establishments located in the United States, regardless of their ownership, but not those located in U.S. territories. (Industrial Production: Construction supplies)

30. IPMINE https://fred.stlouisfed.org/series/IPMINE
Industrial Production - Mining

31. IPN211111GS https://fred.stlouisfed.org/series/IPN211111GS
The industrial production (IP) index measures the real output of all relevant establishments located in the United States, regardless of their ownership, but not those located in U.S. territories. (Industrial Production: Mining: Natural gas)

32. IPN2121S https://fred.stlouisfed.org/series/IPN2121SIPN2121SIPN2121S
The industrial production (IP) index measures the real output of all relevant establishments located in the United States, regardless of their ownership, but not those located in U.S. territories. NAICS = 2121 (Coal mining)

33. IPG211111CS https://fred.stlouisfed.org/series/IPG211111CS
The industrial production (IP) index measures the real output of all relevant establishments located in the United States, regardless of their ownership, but not those located in U.S. territories. NAICS = 211111pt (Crude oil)

34. IPG211111S https://fred.stlouisfed.org/series/IPG211111S
The industrial production (IP) index measures the real output of all relevant establishments located in the United States, regardless of their ownership, but not those located in U.S. territories. NAICS = 211111 (Crude petroleum and natural gas extraction)

35. IPG21222S https://fred.stlouisfed.org/series/IPG21222S
The industrial production (IP) index measures the real output of all relevant establishments located in the United States, regardless of their ownership, but not those located in U.S. territories. NAICS = 21222 (Gold ore and silver ore mining)

36. IPG21223S https://fred.stlouisfed.org/series/IPG21223S
The industrial production (IP) index measures the real output of all relevant establishments located in the United States, regardless of their ownership, but not those located in U.S. territories. NAICS = 21223 (Copper, nickel, lead, and zinc mining)

37. IPN213111S https://fred.stlouisfed.org/series/IPN213111S
The industrial production (IP) index measures the real output of all relevant establishments located in the United States, regardless of their ownership, but not those located in U.S. territories. NAICS = 213111 (Drilling oil and gas wells)

38. IPMAN https://fred.stlouisfed.org/series/IPMAN
Industrial Production: Manufacturing

39. IPG321S https://fred.stlouisfed.org/series/IPG321S
The industrial production (IP) index measures the real output of all relevant establishments located in the United States, regardless of their ownership, but not those located in U.S. territories. NAICS = 321 (Wood product manufacturing)

40. IPG332S https://fred.stlouisfed.org/series/IPG332S
The industrial production (IP) index measures the real output of all relevant establishments located in the United States, regardless of their ownership, but not those located in U.S. territories. NAICS = 332 (Fabricated metal product manufacturing)

41. IPG3361T3S https://fred.stlouisfed.org/series/IPG3361T3S
The industrial production (IP) index measures the real output of all relevant establishments located in the United States, regardless of their ownership, but not those located in U.S. territories. NAICS = 3361; 3362; 3363 (Motor vehicle manufacturing; Motor vehicle body and trailer manufacturing; Motor vehicle parts manufacturing)

42. IPNMAN https://fred.stlouisfed.org/series/IPNMAN
Industrial Production: Nondurable Manufacturing

43. IPG3273S https://fred.stlouisfed.org/series/IPG3273S
The industrial production (IP) index measures the real output of all relevant establishments located in the United States, regardless of their ownership, but not those located in U.S. territories. NAICS = 3273 (Cement and concrete product manufacturing)

44. IPG3311A2S https://fred.stlouisfed.org/series/IPG3311A2S
The industrial production (IP) index measures the real output of all relevant establishments located in the United States, regardless of their ownership, but not those located in U.S. territories. NAICS = 3311; 3312 (Iron and steel mills and ferroalloy manufacturing; Steel product manufacturing from purchased steel)

45. IPCONGD https://fred.stlouisfed.org/series/IPCONGD
Industrial Production: Consumer Goods

46. IPDCONGD https://fred.stlouisfed.org/series/IPDCONGD
Industrial Production: Durable Consumer Goods

47. IPNCONGD https://fred.stlouisfed.org/series/IPNCONGD
Industrial Production: Nondurable Consumer Goods

48. IPFINAL https://fred.stlouisfed.org/series/IPFINAL
Industrial Production: Final Products (Market Group)

49. UEMPMEAN https://fred.stlouisfed.org/series/UEMPMEAN
Average (Mean) Duration of Unemployment. The series comes from the 'Current Population Survey (Household Survey)'.

50. UEMPMED https://fred.stlouisfed.org/series/UEMPMED
Median Duration of Unemployment. The series comes from the 'Current Population Survey (Household Survey)'.

51. MPRIME https://fred.stlouisfed.org/series/MPRIME
Bank Prime Loan Rate. Averages of daily figures. Rate posted by a majority of top 25 (by assets in domestic offices) insured U.S.-chartered commercial banks. Prime is one of several base rates used by banks to price short-term business loans.

52. BUSLOANSNSA https://fred.stlouisfed.org/series/BUSLOANSNSA
Commercial and Industrial Loans, All Commercial Banks

53. CILLCBM027NBOG https://fred.stlouisfed.org/series/CILLCBM027NBOG
Commercial and Industrial Loans, Large Domestically Chartered Commercial Banks

54. CILSCBM027NBOG https://fred.stlouisfed.org/series/CILSCBM027NBOG
Commercial and Industrial Loans, Small Domestically Chartered Commercial Banks

55. CILDCBM027NBOG https://fred.stlouisfed.org/series/CILDCBM027NBOG
Commercial and Industrial Loans, Domestically Chartered Commercial Banks

56. CPIAUCSL https://fred.stlouisfed.org/series/CPIAUCSL
The Consumer Price Index for All Urban Consumers: All Items (CPIAUCSL) is a measure of the average monthly change in the price for goods and services paid by urban consumers between any two time periods. It can also represent the buying habits of urban consumers. This particular index includes roughly 88 percent of the total population, accounting for wage earners, clerical workers, technical workers, self-employed, short-term workers, unemployed, retirees, and those not in the labor force.
The CPIs are based on prices for food, clothing, shelter, and fuels; transportation fares; service fees (e.g., water and sewer service); and sales taxes. Prices are collected monthly from about 4,000 housing units and approximately 26,000 retail establishments across 87 urban areas. To calculate the index, price changes are averaged with weights representing their importance in the spending of the particular group. The index measures price changes (as a percent change) from a predetermined reference date. In addition to the original unadjusted index distributed, the Bureau of Labor Statistics also releases a seasonally adjusted index. The unadjusted series reflects all factors that may influence a change in prices. However, it can be very useful to look at the seasonally adjusted CPI, which removes the effects of seasonal changes, such as weather, school year, production cycles, and holidays.
The CPI can be used to recognize periods of inflation and deflation. Significant increases in the CPI within a short time frame might indicate a period of inflation, and significant decreases in CPI within a short time frame might indicate a period of deflation. However, because the CPI includes volatile food and oil prices, it might not be a reliable measure of inflationary and deflationary periods. For a more accurate detection, the core CPI (Consumer Price Index for All Urban Consumers: All Items Less Food & Energy [CPILFESL]) is often used. When using the CPI, please note that it is not applicable to all consumers and should not be used to determine relative living costs. Additionally, the CPI is a statistical measure vulnerable to sampling error since it is based on a sample of prices and not the complete average.

57. INVCMRMTSPL https://fred.stlouisfed.org/series/INVCMRMTSPL
Real Manufacturing and Trade Inventories (INVCMRMTSPL) was first constructed by the Federal Reserve Bank of St. Louis in June 2013. It is calculated using Real Manufacturing and Trade Inventories (INVHMRMT) (https://fred.stlouisfed.org/series/INVHMRMT) and Real Manufacturing and Trade Inventories (INVCMRMT) (https://fred.stlouisfed.org/series/INVCMRMT).

58. IR https://fred.stlouisfed.org/series/IR
Import Price Index (End Use): All commodities, based on Import/Export Price Indexes web site at http://www.bls.gov/mxp.

59. IQ https://fred.stlouisfed.org/series/IQ
Export Price Index (End Use): All commodities, based on Import/Export Price Indexes web site at http://www.bls.gov/mxp.

60. EXJPUS https://fred.stlouisfed.org/series/EXJPUS
Japan / U.S. Foreign Exchange Rate
Averages of daily figures. Noon buying rates in New York City for cable transfers payable in foreign currencies.
This data series is updated from the source files in the Data Download Program (http://www.federalreserve.gov/datadownload/Choose.aspx?rel=h10). The files are updated on a weekly basis every Monday. If Monday is a holiday, the data files are updated the next business day.
Monthly values are averages of the daily data available. Preliminary value for the current month is provided by the source even if not all daily values are available for the entire month.
Please note that the values reported on the press release may not correspond to the values in the Data Download Program when the press release is published on a day other than Monday. This inconsistency is resolved on the next available weekly release date.

61. M2SL https://fred.stlouisfed.org/series/M2SL
M2 Money Stock
M2 includes a broader set of financial assets held principally by households. M2 consists of M1 plus: (1) savings deposits (which include money market deposit accounts, or MMDAs); (2) small-denomination time deposits (time deposits in amounts of less than $100,000); and (3) balances in retail money market mutual funds (MMMFs). Seasonally adjusted M2 is computed by summing savings deposits, small-denomination time deposits, and retail MMMFs, each seasonally adjusted separately, and adding this result to seasonally adjusted M1.

62. AMBSL https://fred.stlouisfed.org/series/AMBSL
St. Louis Adjusted Monetary Base
This series has been reconstructed starting July 14, 2003. For further information, please refer to https://files.stlouisfed.org/research/publications/review/03/09/Anderson.pdf. Historical data and components are available at https://files.stlouisfed.org/research/publications/review/03/09/0309ra.xls.
Further information and definitions are available at: http://research.stlouisfed.org/publications/mt/.

2 Absence of Seasonality

Seasonally adjusted indicators given by the FRED Economic Data are used if available. Non-seasonally adjusted indicators are 8, 16–18, 51–55, 58–59. However, we verified that seasonality is practically absent in all these time-series. In fact, power-spectrums for the indicators given in Fig.S1, Fig.S2, and Fig.S3 show that there is no significant seasonality in all the indicators at either of quarter, half-year, or one-year.

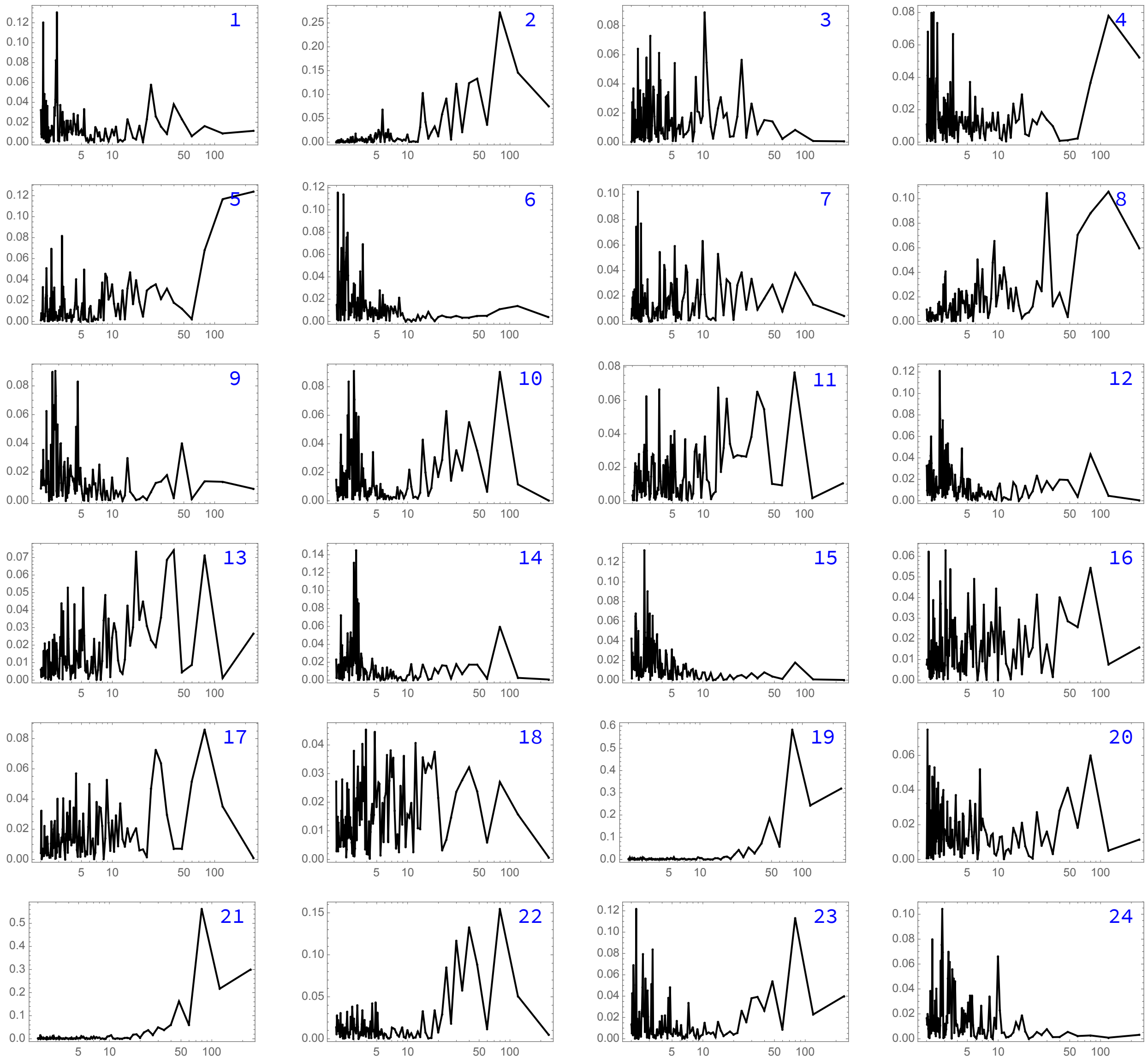

Figure S1. Periodograms of the macro variables collected for this study.

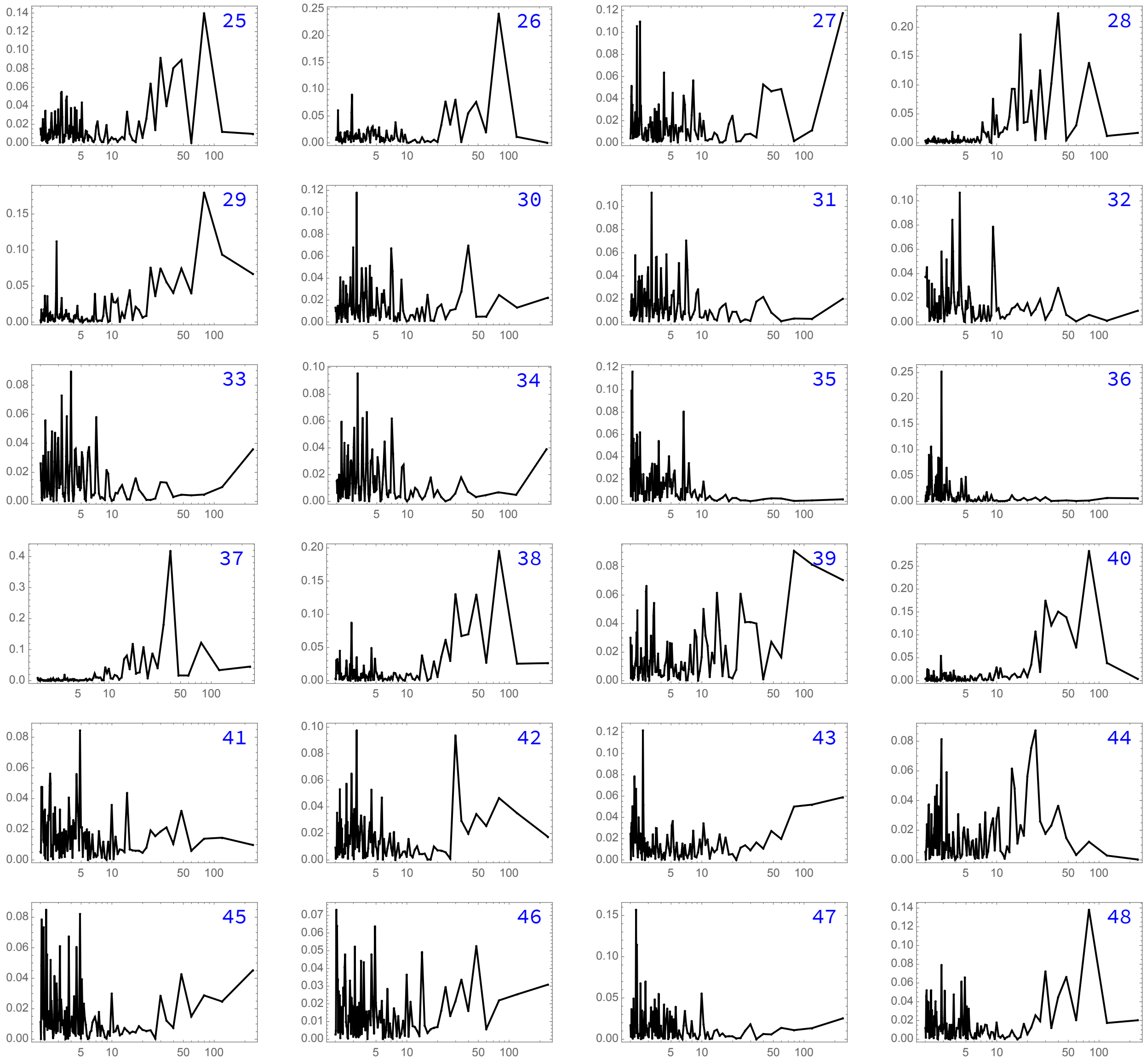

Figure S2. Continuation of Fig.S1.

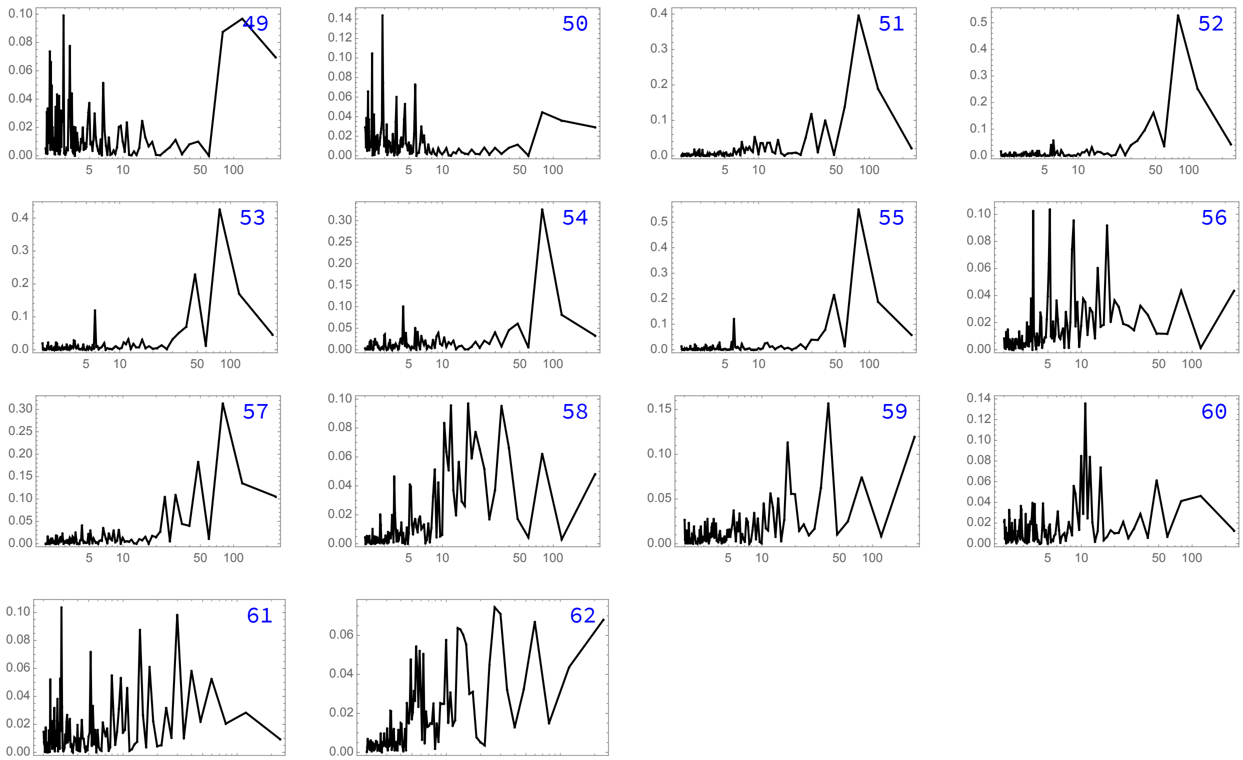

Figure S3. Continuation of Fig.S2.

3 Methods

CHPCA

CHPCA has been applied to data in various fields, such as meteorology/climatology, signal processing, finance and economics[2–6]. In the following, we give a concise summary of this method.

Let us denote the logarithmic difference[1] of the time-series by $w_\alpha(t)$, where $\alpha = 1,2,\cdots N$ is the indicator for the time series (in the current data, $N = 62$), and $t = 1,2,\cdots,T$ is the time in months ($T = 239$). We first complexify each time series using the discrete Fourier decomposition and then doing the following replacement in each component,

$$\cos(\omega t) \to e^{-i\omega t}, \quad \sin(\omega t) \to ie^{-i\omega t}. \tag{1}$$

This leaves the original time-series $w_\alpha(t)$ intact in the real part and the added imaginary part makes the resulting complex time series $\widetilde{w}_\alpha(t)$ rotates in the clock-wise direction on its complex plane. This way, time-shift of $\pi/(2\omega)$ is introduced to the imaginary part, which allows us to go beyond equal-time PCA analysis.

Next, we calculate the complex correlation coefficient

$$\widetilde{C}_{\alpha\beta} := \langle \widetilde{w}_\alpha(t)\widetilde{w}^*_\beta(t)\rangle_t, \tag{2}$$

where $\widetilde{w}_\alpha(t)$ has been standardized with average equal to zero and standard deviation equal to 1 and $\langle\dots\rangle_t$ stands for time average operation. The complex correlation coefficient $\widetilde{C}_{\alpha\beta}$ gives the strength of the correlation between time-series α and β by its absolute value, and the time-delay between them by its phase, which is actually a nonlinear average of time-shift in each Fourier component.

We then obtain the eigenvalues $\tilde{\lambda}^{(n)}$ and the eigenvectors $\widetilde{\boldsymbol{V}}^{(n)}$ for $\widetilde{\boldsymbol{C}} = (\widetilde{C}_{\alpha\beta})$:

$$\widetilde{\boldsymbol{C}}\widetilde{\boldsymbol{V}}^{(n)} = \tilde{\lambda}^{(n)}\widetilde{\boldsymbol{V}}^{(n)}. \tag{3}$$

We note that the followings hold:

$$\widetilde{\boldsymbol{V}}^{(n)\dagger}\cdot\widetilde{\boldsymbol{V}}^{(m)} = \delta_{nm}, \tag{4}$$

and

$$\sum_{n=1}^{N}\tilde{\lambda}^{(n)} = N. \tag{5}$$

We number the eigenvalues and the eigenmodes in descending order of the eigenvalues: $\tilde{\lambda}^{(1)} \geq \tilde{\lambda}^{(2)} \geq \cdots \geq \tilde{\lambda}^{(N)}$.

Each of the complexified time series is decomposed to sum of the eigenmodes:

$$\widetilde{\boldsymbol{w}}(t) = \sum_{\alpha=1}^{N}\widetilde{w}_\alpha(t)\boldsymbol{e}_\alpha = \sum_{\ell=1}^{N}a_\ell(t)\widetilde{\boldsymbol{V}}^{(\ell)}, \tag{6}$$

where $\boldsymbol{e}_\alpha$ is the basis vector of the α-th component, e.g., the transpose of $\boldsymbol{e}_1$ is given by $\boldsymbol{e}_1^{\mathrm{t}} = (1,0,\cdots,0)$. The expansion coefficient $a_\ell(t)$ is called *mode signal* (of the ℓ-th eigenmode). Since the eigenvectors $\widetilde{\boldsymbol{V}}^{(\ell)}$'s form an orthonormal complete basis set, they are given by the following:

$$a_\ell(t) = \widetilde{\boldsymbol{V}}^{(\ell)\dagger}\widetilde{\boldsymbol{w}}(t). \tag{7}$$

The mode signal represents temporal behavior of the eigenmode and its strength is measured by

$$I_\ell(t) = |a_\ell(t)|^2. \tag{8}$$

We note the mutual orthogonality of $\widetilde{\boldsymbol{V}}^{(\ell)}$'s gives the following sum rule:

$$I(t) = \sum_{\ell=1}^{N}I_\ell(t). \tag{9}$$

And taking time average of Eq. (9) yields the sum rule, Eq. (5), for the eigenvalues with $\langle I(t)\rangle_t = N$ and $\langle I_\ell(t)\rangle_t = \tilde{\lambda}_\ell$.

[1] For time series which takes both positive and negative signs, the logarithmic difference is replaced by the simple difference.

RRS

In order to find significant eigenmodes $\widetilde{\boldsymbol{V}}^{(n)}$ that represent statistically significant co-movements (signals), we carry out the significance test by Rotational Random Shuffling (RRS) simulation[6–9]. In this simulation, in order to destroy correlations between time series, each time-series are rotated with its head and the end joined randomly and independently, and then the eigenvalues are calculated. By carrying out this for many times, we obtain the distribution of each eigenvalues. Any eigenvalue above the corresponding RRS distribution is identified to be associated with significant comovements. This way, each auto-correlation is preserved and only the inter-correlation is destroyed, guaranteeing that the difference between the simulated eigenvalue distribution and the true eigenvalue comes from the presence/absence of the inter-correlation.

Using these significant modes, we construct the significant part of the complex correlation matrix:

$$\widetilde{C}^{(\mathrm{sig})}_{\alpha\beta} = \sum_{n=1}^{S} \tilde{\lambda}^{(n)} \widetilde{V}^{(n)}_{\alpha} \widetilde{V}^{(n)*}_{\beta}. \tag{10}$$

where S is the number of significant modes found in the CHPCA. (As is shown in the result section, we have found $S = 6$ in the current case.)

Hodge Decomposition

Hodge decomposition allows one to order nodes from leading to lagging in a directed network. Let us first describe its essence in a manner suitable for our application.

Let us denote the flow from node α to node β by $F_{\alpha\beta}$ in a directed network with N nodes. By this definition, it is anti-symmetric, $F_{\alpha\beta} = -F_{\beta\alpha}$. The weight $e_{\alpha\beta}$ is defined by,

$$e_{\alpha\beta} = \begin{cases} 1 & \text{if } F_{\alpha\beta} \neq 0; \\ 0 & \text{otherwise,} \end{cases} \tag{11}$$

which is symmetric; $e_{\alpha\beta} = e_{\beta\alpha}$.

The Hodge Decomposition aims to decompose the flow $F_{\alpha\beta}$ to two parts:

$$F_{\alpha\beta} = F^{(\mathrm{g})}_{\alpha\beta} + F^{(\mathrm{c})}_{\alpha\beta}. \tag{12}$$

The first term $F^{(\mathrm{g})}_{\alpha\beta}$ is the *gradient flow*, which can be written in the following manner:

$$F^{(\mathrm{g})}_{\alpha\beta} = e_{\alpha\beta} \left(\phi_\alpha - \phi_\beta\right). \tag{13}$$

In the above, ϕ_α is called the *Hodge potential* of the node α, that provides a measure of hierarchy of the nodes. The second term $F^{(\mathrm{c})}_{\alpha\beta}$ $(= -F^{(\mathrm{c})}_{\beta\alpha})$ is the *circular flow* that satisfies the following divergence-free property:

$$\sum_{\beta=1}^{N} F^{(\mathrm{c})}_{\alpha\beta} = 0 \quad (\alpha = 1, \cdots, N). \tag{14}$$

The equations (12)–(14) may be solved as follows: First we rewrite Eq. (14) by using Eq. (12) and Eq. (13):

$$\sum_{\beta=1}^{N} H_{\alpha\beta}\phi_\beta = \sum_{\beta=1}^{N} F_{\alpha\beta} \quad (\alpha = 1, \cdots, N), \tag{15}$$

$$H_{\alpha\beta} = \delta_{\alpha\beta} \sum_{\gamma} e_{\alpha\gamma} - e_{\alpha\beta}. \tag{16}$$

It is straightforward to prove that the matrix $H = \left(H_{\alpha\beta}\right)$ has only one zero-mode: The inner product with an arbitrary vector $\boldsymbol{f} = (f_\alpha)$ is the following:

$$\boldsymbol{f}^{\mathrm{t}} H \boldsymbol{f} = \frac{1}{2} \sum_{\alpha\beta} e_{\alpha\beta} (f_\alpha - f_\beta)^2 \geq 0. \tag{17}$$

This shows that there is only one eigenmode with zero eigenvalue, whose components are all equal, as long as the network is weakly connected. This eigenmode corresponds to the freedom of choosing the baseline of the Hodge potentials. This zeromode is a trivial one, due to the fact that only the differences $\phi_i - \phi_j$ appears in the definitions. Also, if the network is not weakly connected, there is no way of determining hierarchy of the nodes that belong to different connected components, as they should be. Since there are $N-1$ variables, Eq. (15) has always a solution as soon as we fix the baseline of the Hodge potentials. Once we have $\{\phi_1 . \phi_2 . \cdots \phi_N\}$, the circular flow $F^{(\mathrm{c})}_{\alpha\beta}$'s are obtained from Eq. (12).

Synchronization network

This is a network of time-series. Each nodes represent a time-series, and a link represents correlation between two time-series at the end of the links. In order to define this network, we allow link between a time series α and a time series β whose $|\widetilde{C}^{(\mathrm{sig})}_{\alpha\beta}|$ is greater than a threshold $r^{(\mathrm{th})}$. This is because smaller $|\widetilde{C}^{(\mathrm{sig})}_{\alpha\beta}|$ mean small correlation and we need to create links only where the correlation is of some strength.

The key idea in the visualization of this network is, (1) to choose the flow to be the phase;

$$F_{\alpha\beta} = -\theta_{\alpha\beta}, \tag{18}$$

for $\theta_{\alpha\beta} < 0$, so that flow is from the leading node (α) to the lagging node (β), and (2) to use the value of the Hodge potential for each mode as one of their coordinates, say, the vertical coordinate. This way, we can align the nodes (time-series) from leading to lagging vertically. Since we chose the flow by Eq. (18), nodes with small time-difference will have small difference in Hodge potential and will be close to each other in the Hodge coordinate in general. For this to be possible, we choose the threshold $r^{(\mathrm{th})}$ to be the maximal value, which allows the network to be weakly connected (connected when views as an undirected network). The optimization for visualization is done on direction(s) orthogonal to the Hodge axis, by well-known Spring-Charge methods.

Clustering Analysis

We can resort to percolation model[10] for identifying clusters in the data set. A cluster is a set of changes which are linked because they are "similar". The degree of similarity is defined as follows. First, the data is placed on a square lattice (see Fig. S4). The first and second neighbors of each site are candidates for linkage. We measure the strength $g_{\alpha\beta}$ of coupling between the variable α at time t and price β at time t' ($t' = t$ or $t' = t \pm 1$) by geometric mean of their monthly changes $w_\alpha(t)$ and $w_\beta(t')$:

$$g_{\alpha\beta}(t,t') = \sqrt{w_\alpha(t) w_\beta(t')}\ . \tag{19}$$

The two neighboring variables are regarded as being *linked* if their coupling constant is larger than a certain threshold g_c:

$$g_{\alpha\beta}(t,t') > g_c\ . \tag{20}$$

Identification of clusters depends crucially on the choice of g_c: If we adopt a large value of g_c, variables would fragment to a number of tiny pieces. On the other hand, if it is too small, most variables would be connected to each other and they form a single group. If we carefully adjust g_c close to the percolation threshold in this lattice system, various scales of clusters are formed with a power law distribution. Near the percolation threshold, we can thereby extract information on the clustering properties of the variables in the most effective way. This algorithm for detecting price clusters is illustrated in Figure S4.

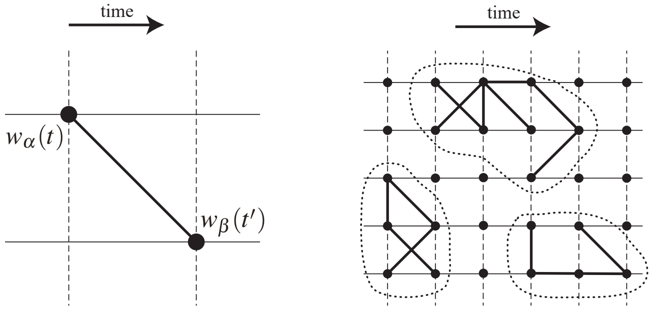

Figure S4. Illustration of the cluster detection. This diagram illustrates the algorithmic way explained in the text for detecting price clusters. The whole data is placed on lattice, where each time series are placed on solid horizontal lines with discreet time proceeding from left to right (vertical dashed lines). Left panel: The thick link between a pair of data is placed if the condition Eq. (20) is met. Right panel: The data points that are connected by links form clusters. In this example, three clusters surrounded by dotted lines are observed.

4 CHPCA results

4.1 Eigenvalues

The result for the distribution of eigenvalues is shown in Figure S5(a) together with that predicted by the RMT. The five largest eigenvalues are found beyond the upper limit of the RMT eigenvalues, the most conservative test to identify significant eigenvalues. Figure S5(b) is a parallel (rank-by-rank) comparison between the eigenvalues for the original data and those for the RRS-processed data. Statistical variation of the RRS eigenvalues were evaluated with 10,000 samples. Here, the mean $+3\sigma$ of the RRS eigenvalues is used as a criterion for significant eigenvalues. This provides us with a more accurate significance test. With this criterion, we identify the six largest eigenvalues as being statistically significant.

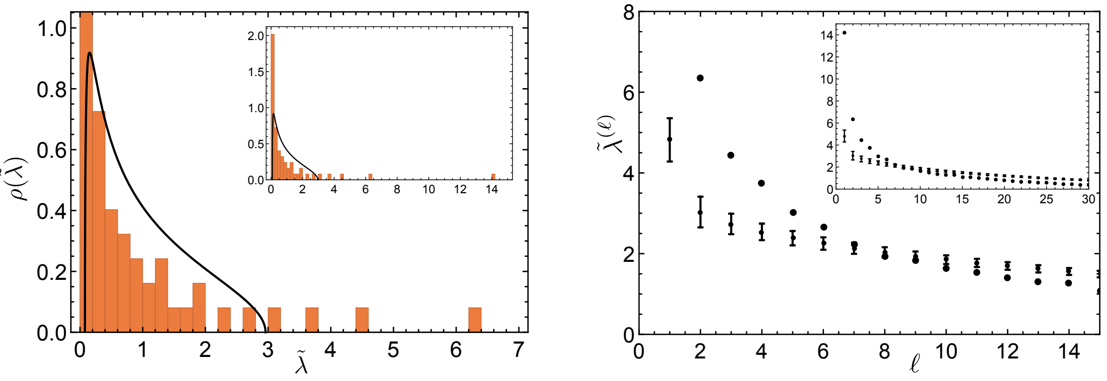

Figure S5. Distribution of the eigenvalues of the CHPCA. Left Panel: Comparison of the distribution with the corresponding result predicted by RMT (solid curve). Right Panel: Parallel comparison of the eigenvalues with those for the RRS-processed data, where the error bars show the 3σ deviation of the RRS results calculated with 10000 samples. The inset in each panel gives an overall view of the comparison.

4.2 The Significant Eigenvectors

Figure S6 shows two most significant eigenvectors associated with the first and second largest eigenvalues in the complex plane representation. The main characteristics of the first eigenvector is the localization of the phases of indicators, which indicates coherence and thus the existence of the economic cycles, as was discussed in the main body of this paper. In the second eigenmode the major players are the indicators of industrial production for energy resources such as crude oil (#30, #31, #33, #34). This implies that these eigenmodes are due to specific economic events, as was discussed in the main body of this paper in terms of the mode signals.

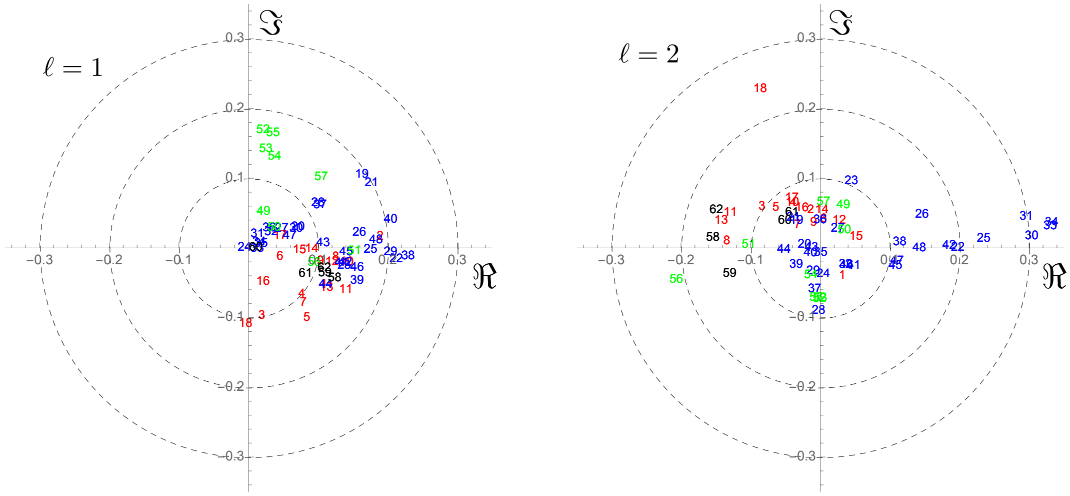

Figure S6. Eigenvectors associated with the first and second largest eigenvalues, depicted in the complex plane. The data numbers given in Table 1 are used as plot markers. The red numbers represent the leading indicators; the blue ones, the coincident indicators; the green ones, the lagging indicators; the black ones, the rest of the macro indicators.

Figure S7 displays the eigenvectors corresponding to the third through sixth largest eigenvalues on the complex plane. The relative intensity of the mode signals associated with those eigenmodes are also shown in Fig. S8. We find no prominent peak in each panel of Fig. S8, such peaks in the first and the second eigenmodes as observed at the time of the recession due to the Lehman crisis and the oil related shocks, respectively.

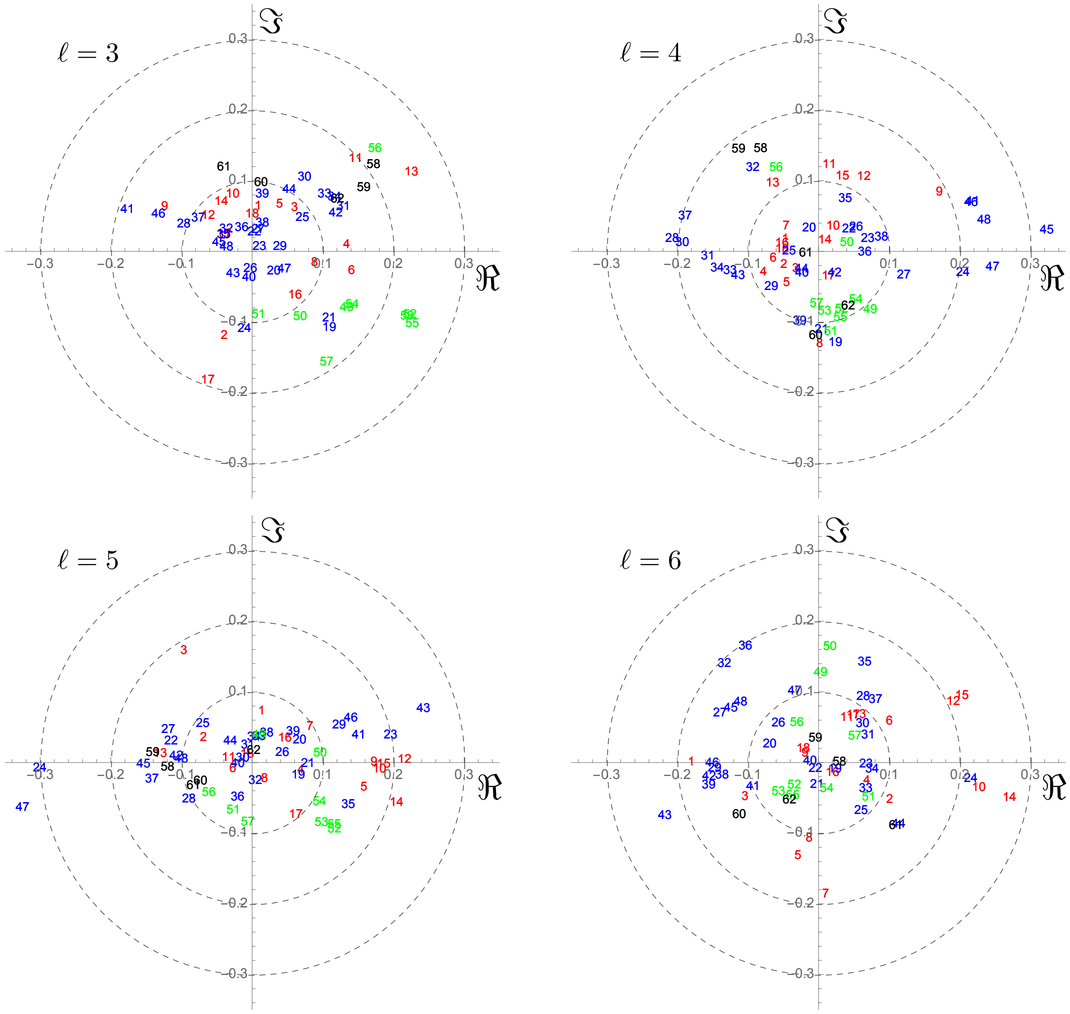

Figure S7. Eigenvectors corresponding to the third through sixth largest eigenvalues in the complex plane representation. The data numbers given in Table 1 are used as plot markers and the color coding for them is the same as in Fig. S6.

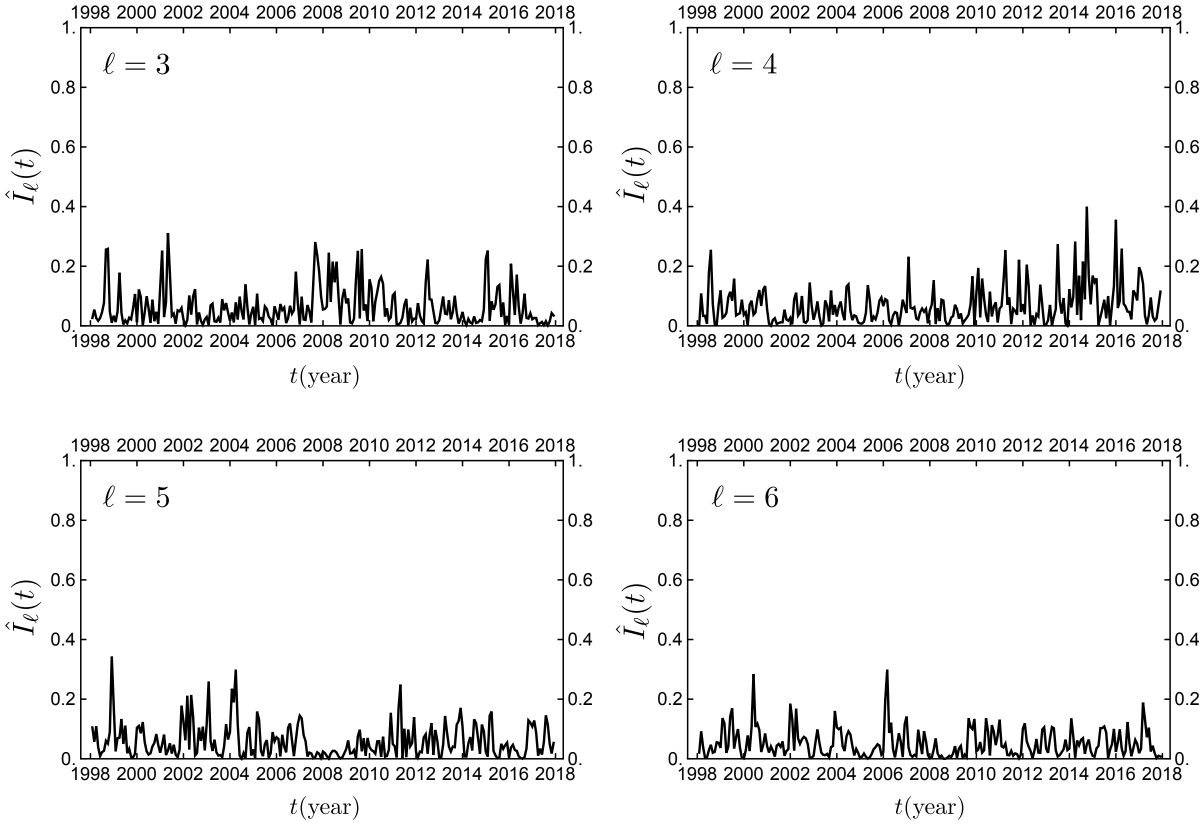

Figure S8. Temporal variation of the relative mode intensity for the third through sixth eigenmodes.

4.3 Comparison with the PCA

Finally, we demonstrate how superior the CHPCA is to the ordinary PCA. This is a question which may naturally come to mind for the readers of this paper.

Corresponding to the complex correlation coefficient, Eq. (2), in the CHPCA, the PCA begins with the real correlation coefficient defined by

$$C_{\alpha\beta} := \langle w_\alpha(t) w_\beta(t) \rangle_t. \tag{21}$$

In parallel to Eq. (3) in the CHPCA, the PCA is mathematically formulated as an eigenvalue problem of the real correlation matrix $\boldsymbol{C} = (C_{\alpha\beta})$:

$$\boldsymbol{C}\boldsymbol{V}^{(n)} = \lambda^{(n)} \boldsymbol{V}^{(n)}. \tag{22}$$

We note that the eigenvalue $\lambda^{(n)}$ is positive definite as well as $\tilde{\lambda}^{(n)}$. On the other hand, components of the eigenvector $\boldsymbol{V}^{(n)}$ are real while those of $\widetilde{\boldsymbol{V}}^{(n)}$ are complex. According to the parallel analysis as shown in Fig. S9, the eight largest eigenvalues are statistically meaningful in contrast to six in the CHPCA. Figure S10 displays the eigenvectors associated with the significant eigenvalues in the PCA. The first eigenvector $\boldsymbol{V}^{(1)}$ clearly indicates that there exists a collective movement of the economic indicators as $\widetilde{\boldsymbol{V}}^{(1)}$ does. Since $\boldsymbol{V}^{(1)}$ is a real vector, however, $\boldsymbol{V}^{(1)}$ does not allow us to extract information on dynamical correlations such as lead-lag relations among those variables; $\boldsymbol{V}^{(1)}$ simply tells us to what extent each of the components contribute to the economic comovement.

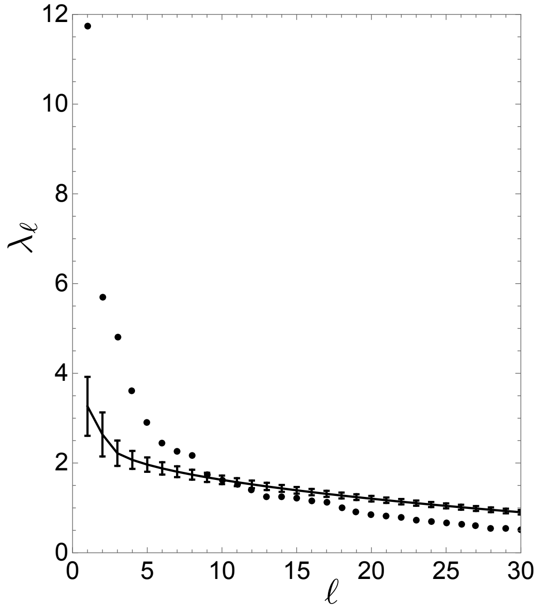

Figure S9. Parallel comparison of the eigenvalues in the PCA with those in the RRS, corresponding to the right panel in Fig. S5.

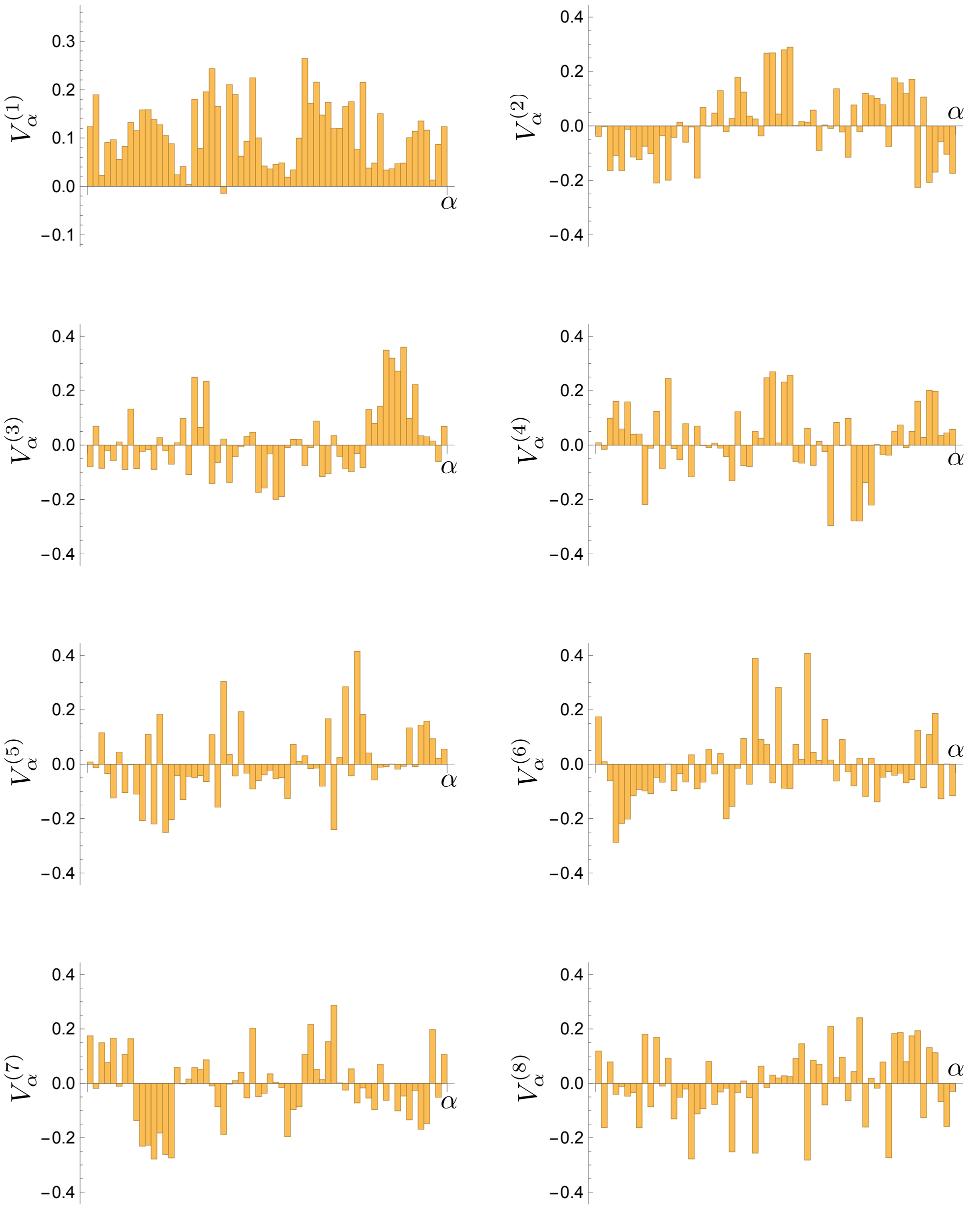

Figure S10. Eigenvectors in the PCA associated with the eight largest eigenvalues, which are statistically significant according to the parallel analysis in Fig. S9. The bars represent values of the individual components of each eigenvector arranged from left to right according to their numbering scheme.

In order to make an explicit connection of the eigenvectors of CHPCA with those of PCA, we define a cosine similarity between $\widetilde{\boldsymbol{V}}^{(\ell)}$ and $\boldsymbol{V}^{(m)}$ as

$$\eta_{\ell,m} = \frac{\Re'[\widetilde{\boldsymbol{V}}^{(\ell)}] \cdot \boldsymbol{V}^{(m)}}{\|\Re'[\widetilde{\boldsymbol{V}}^{(\ell)}]\|} \,, \tag{23}$$

with

$$\Re'[\widetilde{\boldsymbol{V}}^{(\ell)}] = \Re[e^{-i\phi}\widetilde{\boldsymbol{V}}^{(\ell)}] \,. \tag{24}$$

Here $\Re'$ denotes an optimized real axis for projection of $\widetilde{\boldsymbol{V}}^{(\ell)}$. Its rotational angle ϕ relative to the original real axis $\Re$ is determined by maximizing the cosine similarity $\eta_{\ell,m}$. If $\eta_{\ell,m} \simeq 1$, $\widetilde{\boldsymbol{V}}^{(\ell)}$ projected onto the optimized real axis closely resembles $\boldsymbol{V}^{(m)}$. Table 1 gives the results for $\eta_{\ell,m}$ thus calculated for every pair of the significant eigenvectors of CHPCA and those of PCA. In the table, pairs of the eigenvectors with extraordinarily large similarity, $\eta_{\ell,m} > 0.6$, are emphasized in terms of bold type figures. If we adopt such a null model that a pair of normalized real vectors are randomly oriented in 62 dimensions, the threshold for $\eta_{\ell,m}$ indicates that the associated p value is 2.02×10^{-7} ($p = 3.01 \times 10^{-5}$ for $\eta_{\ell,m} = 0.5$). Table 2 lists the rotational angle ϕ of the optimized real axis for each pair of the eigenvectors of CHPCA and PCA, corresponding the cosine similarity values as shown in Table 1.

Reading Table 1 diagonally, we find that each of the eigenvectors of CHPCA has a highly intimate partner among the eigenvectors of PCA. For instance, $\widetilde{\boldsymbol{V}}^{(1)}$ projected onto the optimized real axis which is almost identical to the original real axis ($\phi \simeq 0$) is virtually indistinguishable from $\boldsymbol{V}^{(1)}$. The vectors $\widetilde{\boldsymbol{V}}^{(2)}$ and $\boldsymbol{V}^{(2)}$ are closely connected to each other, and so forth. As an important fact, we remark that $\widetilde{\boldsymbol{V}}^{(1)}$ projected onto the alternative optimized axes, which are almost identical to the original imaginary axis ($\phi \simeq 90$ degrees), resemble $\boldsymbol{V}^{(3)}$. We thus see that partial information on the dynamical correlations between macroeconomic indicators as simply manifested in $\widetilde{\boldsymbol{V}}^{(1)}$ are also reflected on $\boldsymbol{V}^{(2)}$ and $\boldsymbol{V}^{(3)}$. Figure S11 displays the real and imaginary parts of $\widetilde{\boldsymbol{V}}^{(1)}$ to compare with $\boldsymbol{V}^{(1)}$, and $\boldsymbol{V}^{(2)}$ and $\boldsymbol{V}^{(3)}$, respectively, in Fig. S10. Methodologically, at first sight, it may be thought that there is no much difference between CHPCA and PCA. However, it is a kind of the situation that one knows the solution of a problem from the outset. One could hardly reach the whole picture of the comovement of indicators with the results of PCA alone; imagine how to construct $\widetilde{\boldsymbol{V}}^{(1)}$ out of $\boldsymbol{V}^{(1)}$, $\boldsymbol{V}^{(2)}$ and $\boldsymbol{V}^{(3)}$. The eigenmodes of PCA are generally thought to be independent of each other. Such a general idea is not true at all. In fact, there is a possibility that some of the eigenmodes of PCA are dynamically connected to each other to form a single eigenmode of CHPCA. As a by-product of the present comparison between the eigenvectors of CHPCA and PCA, we find that $\widetilde{\boldsymbol{V}}^{(1)}$ is related to $\widetilde{\boldsymbol{V}}^{(2)}$ and $\widetilde{\boldsymbol{V}}^{(3)}$ through $\boldsymbol{V}^{(2)}$ and $\boldsymbol{V}^{(3)}$, respectively. Such similarity of $\widetilde{\boldsymbol{V}}^{(1)}$ and $\widetilde{\boldsymbol{V}}^{(2)}$ or $\widetilde{\boldsymbol{V}}^{(3)}$ indicates that switching between the first and second (third) eigenmodes can take place in a smooth way. Another example of the hidden correspondence between CHPCA and PCA is observable for $\widetilde{\boldsymbol{V}}^{(3)}$. Two orthogonal aspects of $\widetilde{\boldsymbol{V}}^{(3)}$ are well described by $\boldsymbol{V}^{(3)}$ and $\boldsymbol{V}^{(4)}$; the two optimized projection axes cross at nearly right angles. Also we see that $\widetilde{\boldsymbol{V}}^{(3)}$ is related to $\widetilde{\boldsymbol{V}}^{(4)}$ through $\boldsymbol{V}^{(4)}$.

From the above results, we conclude that the CHPCA is much more advantageous than the PCA for investigation of dynamical correlations involved in complex systems including economic cycles.

Table 1. Optimized cosine similarity $\eta_{\ell,m}$, Eq. (23), between the ℓ-th eigenvector in the CHPCA and the m-th eigenvector in the PCA. The results larger than 0.6, the p value of which takes 2.02×10^{-7} for a null model that a pair of normalized real vectors are randomly oriented in 62 dimensions, are shown in bold type.

		PCA (m)							
		1	2	3	4	5	6	7	8
CHPCA (ℓ)	1	**0.996**	**0.634**	**0.717**	0.081	0.039	0.165	0.126	0.029
	2	0.277	**0.888**	0.544	0.170	0.224	0.518	0.043	0.237
	3	0.337	0.354	**0.773**	**0.757**	0.281	0.304	0.141	0.372
	4	0.142	0.284	0.485	**0.820**	0.333	0.522	0.578	0.359
	5	0.194	0.127	0.581	0.145	**0.892**	0.182	0.362	0.387
	6	0.076	0.192	0.052	0.275	0.326	0.386	**0.842**	0.495

Table 2. The rotational angle ϕ (in units of degree) of the projection axis for significant eigenvectors in the CHPCA which optimizes the cosine similarity of them to significant eigenvectors in the PCA, corresponding to the results in Table 1.

		PCA (m)							
		1	2	3	4	5	6	7	8
CHPCA (ℓ)	1	0.7	92.4	88.8	−99.9	−92.1	83.8	91.2	165.0
	2	72.4	−30.4	−130.0	81.8	−92.5	−92.6	97.3	−88.5
	3	45.7	−76.5	−60.5	31.9	21.7	149.0	72.4	58.0
	4	24.2	−84.8	−87.6	−176.0	4.2	122.0	80.5	72.5
	5	72.7	−93.6	−84.4	139.0	−178.0	−171.0	−91.1	60.6
	6	169.0	93.1	−119.0	−41.8	134.0	115.0	44.4	169.0

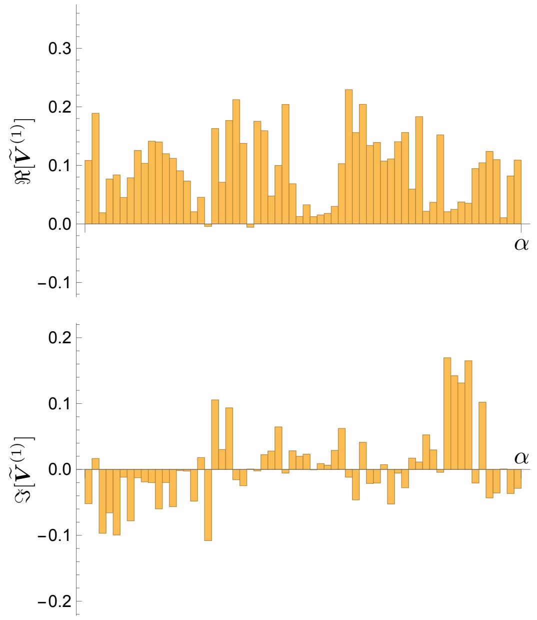

Figure S11. Real (upper panel) and imaginary (lower panel) part of the first eigenvector of CHPCA, compared with the first and third eigenvectors, respectively, in Fig. S10.

References

1. Federal Reserve Bank of St. Louis, FRED Economic Data. https://fred.stlouisfed.org/. Accessed: November, 2018.
2. Gabor, D. Theory of communication. *J. Inst. Electr. Eng.–Part III, Radio Commun. Eng.* **93**, 429–457 (1946).
3. Granger, C. W. J. & Hatanaka, M. *Spectral analysis of economic time series.* (Princeton Univ. Press., 1964).
4. Rasmusson, E. M., Arkin, P. A., Chen, W.-Y. & Jalickee, J. B. Biennial variations in surface temperature over the United States as revealed by singular decomposition. *Mon. Wea. Rev.* **109**, 587–598 (1981).
5. Barnett, T. Interaction of the monsoon and pacific trade wind system at interannual time scales part i: the equatorial zone. *Mon. Weather. Rev.* **111**, 756–773 (1983).
6. Aoyama, H. *et al.* *Macro-Econophysics –New Studies on Economic Networks and Synchronization* (Cambridge University Press, 2017).
7. Iyetomi, H. *et al.* What causes business cycles? –Analysis of the Japanese industrial production data. *J. Jpn. Int. Econ.* **25**, 246–272 (2011).
8. Iyetomi, H. *et al.* Fluctuation-dissipation theory of input-output interindustrial relations. *Phys. Rev. E* **83**, 016103 (2011).
9. Arai, Y., Yoshikawa, T. & Iyetomi, H. Complex principal component analysis of dynamic correlations in financial markets. *Front. Artif. Intell. Appl.* **255**, 111 – 119 (2013). DOI 10.3233/978-1-61499-264-6-111.
10. Kirkpatrick, S. Percolation and conduction. *Rev. Mod. Phys.* **45**, 574–588 (1973). URL https://link.aps.org/doi/10.1103/RevModPhys.45.574. DOI 10.1103/RevModPhys.45.574.
